# Tumor methionine metabolism drives T-cell exhaustion in hepatocellular carcinoma

**DOI:** 10.1038/s41467-021-21804-1

**Published:** 2021-03-05

**Authors:** Man Hsin Hung, Joo Sang Lee, Chi Ma, Laurence P. Diggs, Sophia Heinrich, Ching Wen Chang, Lichun Ma, Marshonna Forgues, Anuradha Budhu, Jittiporn Chaisaingmongkol, Mathuros Ruchirawat, Eytan Ruppin, Tim F. Greten, Xin Wei Wang

**Affiliations:** 1 Laboratory of Human Carcinogenesis, Center for Cancer Research, National Cancer Institute, Bethesda, Maryland USA; 2 Cancer Data Science Lab, National Cancer Institute, National Institutes of Health, Bethesda, Maryland USA; 3 Gastrointestinal Malignancy Section, Thoracic and Gastrointestinal Oncology Branch, Center for Cancer Research, National Cancer Institute, National Institutes of Health, Bethesda, MD 20892 USA; 4 Liver Cancer Program, Center for Cancer Research, National Cancer Institute, Bethesda, Maryland USA; 5 Laboratory of Chemical Carcinogenesis, Chulabhorn Research Institute, Bangkok, Thailand; 6 Center of Excellence on Environmental Health and Toxicology, Office of Higher Education Commission, Ministry of Education, Bangkok, Thailand

**Keywords:** Cancer metabolism, Cancer microenvironment, Hepatocellular carcinoma, Immunoediting

## Abstract

T-cell exhaustion denotes a hypofunctional state of T lymphocytes commonly found in cancer, but how tumor cells drive T-cell exhaustion remains elusive. Here, we find T-cell exhaustion linked to overall survival in 675 hepatocellular carcinoma (HCC) patients with diverse ethnicities and etiologies. Integrative omics analyses uncover oncogenic reprograming of HCC methionine recycling with elevated 5-methylthioadenosine (MTA) and S-adenosylmethionine (SAM) to be tightly linked to T-cell exhaustion. SAM and MTA induce T-cell dysfunction in vitro. Moreover, CRISPR-Cas9-mediated deletion of MAT2A, a key SAM producing enzyme, results in an inhibition of T-cell dysfunction and HCC growth in mice. Thus, reprogramming of tumor methionine metabolism may be a viable therapeutic strategy to improve HCC immunity.

## Abstract (web summary)

Intratumoral CD8+ T cells commonly display a dysfunctional state, however it remains unclear whether tumor cell metabolism actively promotes T-cell exhaustion. Here, the authors show that the tumor methionine recycling pathway has a central role in promoting T-cell dysfunction in hepatocellular carcinoma, contributing to tumor immune evasion.

## Introduction

Immune cells are an important component of the tumor microenvironment (TME), which has critical roles in determining tumor progression^1,2^. CD8+ T cells are a major immune cell type that exerts antitumor activity. However, intratumoral CD8+ T cells commonly display a dysfunctional state known as exhausted T-cell that excludes them from effectively eliminating cancer cells^3^. Prolonged antigen exposure and sustained inflammatory stimulations have been considered as potential mechanisms driving T cells from an effective state to an exhausted state^4^. Whether tumor cells actively promote the establishment and progression of T-cell exhaustion and to what extent has remained a key open question. It is known that cancer cells often show a high dependence on an altered metabolic state for stress adaptation and tumor cell proliferation^5^. Intriguingly, recent emerging evidence suggests that metabolic changes in tumor cells also affect the composition and function of the non-cancer cells residing in the same microenvironment^6,7^.

Hepatocellular carcinoma (HCC) is the fourth most lethal type of malignancies worldwide and effective treatment for advanced HCC is currently lacking^8^. Exhaustion of infiltrating T cells has been convincingly observed in HCC, and has been linked to poor clinical outcome^9^. However, what induces T-cell exhaustion in HCC and whether tumor metabolism drives T-cell dysfunction has not been well characterized.

In this study, we aim to study these questions systematically by profiling the bulk genome, transcriptome, and metabolome as well as single-cell transcriptome. We perform ATAC sequencing and other functional assays to assess the state of T cells and HCC development. Our results reveal the central role of tumor methionine recycling pathway as a driver of T-cell dysfunction in HCC, leading tumor immune evasion. Consequently, targeting the methionine salvage pathway may represent a future attractive strategy to prevent and revert T-cell exhaustion thereby improving immune surveillance in HCC and potentially enhancing patients’ response to checkpoint therapy.

## Results

### Transcriptome defined T-cell exhaustion status is prognostic in HCC

**Fig. 1:**
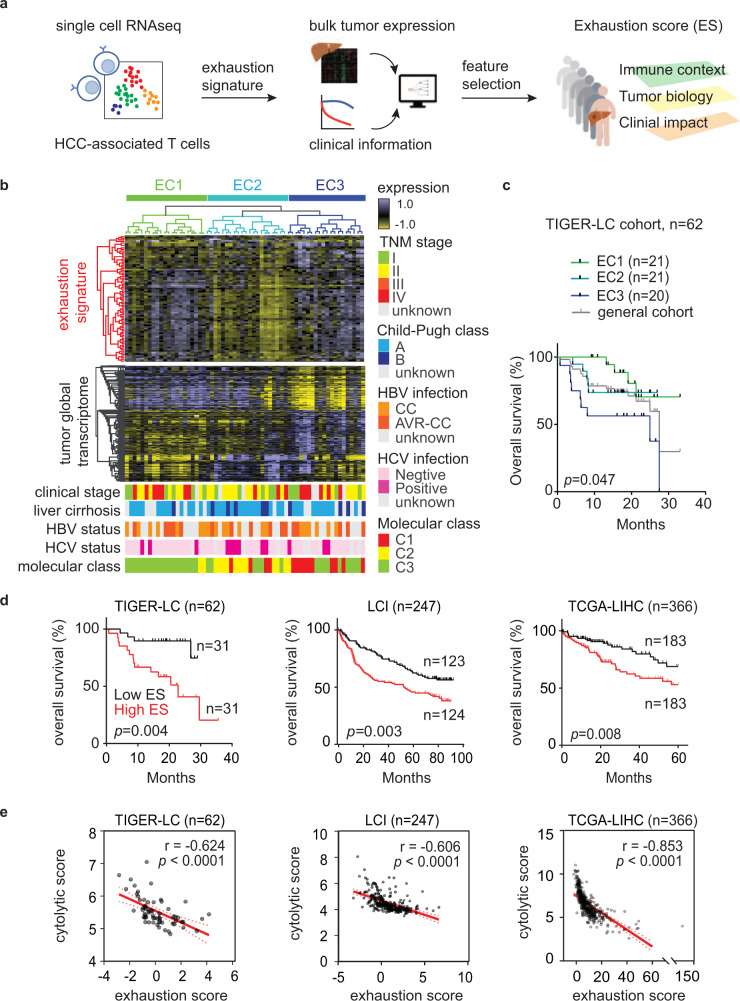
Identification of exhaustion score that models CD8+ T-cell dysfunction in HCC. **a** Study overview. **b** The expression of T-cell exhaustion signature links to tumor transcriptome and patient survival. The upper heatmap reveals the results of unsupervised hierarchical clustering of tumor samples (TIGER-LC cohort, *N* = 62) based on 82 genes associated with exhausted CD8 T cells (Supplementary Data 1) and the lower heatmap shows the most variable genes (*n* = 1533, Supplementary Data 2), and important clinical and molecular characters associated with exhaustion signature among tumor samples. According to the expressions of T-cell exhaustion genes, patients are divided into three different exhaustion clusters (ECs). **c** Kaplan–Meier survival analysis of the 62 HCC patients based on the ECs with two-sided log-rank *p* value. The survival curve of the overall cohort was shown here (gray) but was not included for the calculation of *p* value. **d** Exhaustion score predicts HCC patient survival. Patients from TIGER-LC cohort, LCI cohort, and TCGA-LIHC cohort are stratified by the median value of exhaustion score in each cohort, and the results of Kaplan–Meier survival analysis are shown here and the survival significance is determined using a two-sided log-rank test. **e** The correlation of exhaustion score and cytolytic score in HCC tumors. Correlation coefficient and *P* values are based on two-sided Spearman’s rank correlation coefficient test. Source data are provided as a Source Data file.

To determine the degree of T-cell exhaustion in bulk tumor samples, we analyzed the single-cell transcriptome data of 5063 HCC-associated T cells^10^ (GSE98638) and identified 82 T-cell exhaustion-specific genes (Supplementary Data 1). Hierarchical analysis of the bulk expression of HCC tumors from the TIGER-LC cohort described previously^11^ using these exhaustion-specific genes revealed three main clusters, and the EC3 cluster with activation of most of the T-cell exhaustion genes were associated with the worse clinical outcome and increasing infiltration of cells with immunosuppressive properties, such as Tregs and M2 macrophages (Fig. 1a–c and Supplementary Fig. 1a). Notably, such an association was observed only in tumors but not in adjacent non-tumor tissues (Supplementary Fig. 1b) and was independent of other known prognostic factors in HCC (Fig. 1b and Supplementary Table 1). The tumor subgroups associated with T-cell exhaustion-specific genes display unique tumor transcriptomes (Fig. 1b, Supplementary Data 2), suggesting a role of tumor biology in affecting the function of T cells in TME.

Among the T-cell exhaustion-specific genes, the combination of *YARS*, *TPI1*, *PKM*, and *MTHFD2* best differentiates the survival of HCC patients based on a Cox proportional hazards model (Fig. 1a) (Supplementary Table 2). This analysis also yields a weighted exhaustion score (ES) that has enabled us to infer the degree of T-cell exhaustion from bulk expression in each HCC sample. Consistently, the ES values were positively correlated with the collective expression of T-cell exhaustion-specific genes, but further improve the prediction of patients’ survival (Supplementary Fig. 2a, b). Since the ES was obtained from the TIGER-LC cohort as a training set, we studied its robustness using several additional, ethnically different HCC patient cohorts. We found consistent predictive power in both 247 Chinese patients (LCI cohort) and 366 American patients (TCGA cohort) (Fig. 1d). A multivariant Cox regression analysis revealed that the ES predicts HCC survival [Hazard ratio =  3.27 (95% confidence interval = 1.85–5.79), *p* < 0.001] independent of common molecular subtypes, tumor staging, and cirrhosis status in the TIGER-LC cohort^11^ (Supplementary Table 3). Importantly, the ES was inversely associated with immune cytolytic activity (CYT) based on the expression of *GZMA* and *PRF1*, the two key cytolytic effectors^12^, in all three cohorts (Fig. 1e), indicating that tumors with a high ES indeed have a T-cell dysfunction. Notably, the inverse correlation of ES and CYT was also observed in matched non-tumor liver tissues from all three HCC cohorts and liver tissues from chronic hepatitis C infected patients, but not in liver tissues with several acute infection/inflammation-induced conditions (Supplementary Fig. 2c–f). As a majority of HCCs arise in the context of chronic inflammation^13^, the above results indicated that the negative association of T-cell exhaustion and CYT is specifically related to a chronic inflammatory state, consistent with the notion that T-cell exhaustion is a specific form of T-cell hypofunction induced by chronic inflammatory stimulation^4^.

### Tumor methionine metabolism and T-cell exhaustion

We performed pathway analysis on the genes that showed a significant correlation with ES for the investigation of potential mechanisms contributing to T-cell dysfunction in HCC. Interestingly, in addition to many immune-associated signaling pathways, we found that metabolism-related pathways, such as glycolysis and methionine degradation, showed significant changes with the progression of ES, suggesting that alteration of tumor metabolism may have a role in reprogramming T-cell function in HCC (Supplementary Table 4).

**Fig. 2:**
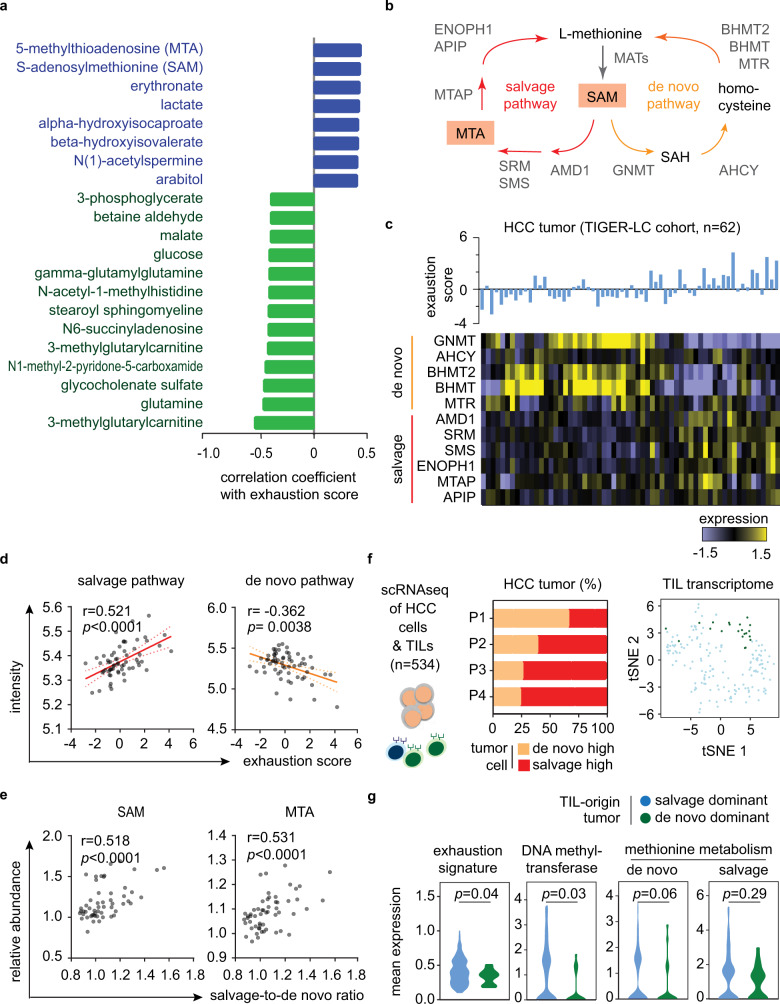
Dysregulation of methionine recycling machinery leads to increasing tumor SAM and MTA content and drives T-cell exhaustion in HCC Tumor. **a** The waterfall plot demonstrates the correlation between ES and the tumor-to-non-tumor level of different metabolites in HCC tumors of TIGER-LC cohort (Supplementary Table 5). **b** Schema of methionine recycling machinery. **c** Heatmap reveals the expressions of genes involving methionine recycling pathways (lower panel) and the associated tumor ES (upper panel) in HCC tumors. **d** The relationship of ES and the expressions of salvage pathway and de novo pathway in HCC tumors. (*n* = 62) Correlation coefficient and *P* values are based on two-sided Spearman’s rank correlation coefficient test. **e** The relationships of the tumor SAM and MTA contents with the salvage-to-de novo ratio. (*n* = 62) Correlation coefficient and *P* values are assessed by two-sided Spearman’s rank correlation coefficient test. **f**, **g** Single-cell transcriptomic study on HCC tumors validates the metabolic interaction linking cancer methionine metabolism and T-cell exhaustion. Single-cell transcriptome of malignant cells and associated T cells are obtained from four HCC patients (GEO125449, *n* = 534). We defined malignant cells as salvage-high or de novo-high by the mean value of the salvage-to-de novo ratio of all malignant cells. We then reconstructed each tumor according to the proportion of salvage-high (colored in red) or de novo-high (colored in orange) cancer (**f**, left panel). According to the dominant methionine metabolic status of HCC cells, tumors from P3 and P4 are defined as salvage-dominant tumors and P1 tumor is considered as de novo-dominant tumor. 175T cells associated with the above-mentioned tumors are identified. t-SNE plot showed the transcriptome differences among T cells originated from salvage-dominant tumors to de novo-dominant tumor (**f**, right panel). The expressions of T-cell exhaustion-specific genes, DNA methyltransferase genes, and methionine metabolic genes of T cells were examined and summarized using violin plots **g**. Statistical significance is determined by two-sided independent *t* test. Source data are provided as a Source Data file.

To better define T-cell-dysfunction-related tumor metabolism, we analyzed tumor-specific metabolomic data from the TIGER-LC cohort^11^. Among 718 detected metabolites, we found 21 metabolites that were significantly correlated with the ES (FDR < 0.05; Fig. 2a, Supplementary Table 5), and several of them, like, lactate^14^ and glutamine^15^, were reported to influence the function of tumor-infiltration T cells, supporting the ability of current strategy on identifying critical tumor metabolites related to T-cell biology. The top-2 metabolites belong to the methionine metabolism pathway, namely, 5-methylthioadenosine (MTA, *ρ* = 0.444, Spearman correlation test, *p* = 0.000685) and S-adenosylmethionine (SAM, *ρ* = 0.437, Spearman correlation test, *p* = 0.000841). We found that the levels of SAM and MTA were significantly higher in tumors than paired non-tumor tissues in both TIGER-LC cohort and a subset of patients from LCI cohort with available metabolomic data^16^ (Supplementary Fig. 3a). Higher SAM/MTA levels, as well as high ES level, were negatively correlated with the expressions of most of the genes involving cytokine- and chemokine-signaling in tumor tissues, whereas CYT levels showed an opposite relationship (Supplementary Fig. 3b and Supplementary Data 3). Notably, *TGFBR1*, encodes for transforming growth factor-β signaling receptor, and *CCL28*, chemokine involves lymphocyte trafficking, had the highest positive associations with higher T-cell exhaustion or MTA/SAM levels, whereas genes involved in T-cell differentiation and activation, such as *CXCR3, CXCR6,* and *CCR7*, had the highest negative association. Tumors SAM/MTA level was positively associated with tumor ES (Supplementary Fig. 3c). The expression levels of immune checkpoint genes that negatively regulate T-cell activation, such as *PDCD1* and *HAVCR2*, were significantly increased in SAM/MTA-abundant tumors, whereas the expression of ICOS, an immune checkpoint that promote T-cell activation, was not related to tumor SAM/MTA levels (Supplementary Fig. 3d).

### Oncogenic activation of methionine salvage pathway-related genes

SAM and MTA are a part of the methionine salvage pathway, a major methionine recycling mechanism to replenish the methionine pool (Fig. 2b)^17^. To determine how SAM/MTA levels are elevated in T-cell exhaustion dominant tumors, we examined the expression levels of genes in both salvage- and de novo pathways in HCC tumors. We found that these pathways tend to be inversely expressed in HCC tumors, that is the upregulation of the de novo pathway is typically accompanied by downregulation of the salvage pathway, or vice versa (Fig. 2c). Furthermore, T-cell exhaustion levels were positively correlated with salvage pathway expression and negatively correlated with de novo pathway expression (Fig. 2d). We calculated the ratio of salvage pathway expression to de novo pathway expression to model the changes of methionine recycling and found that the levels of SAM/MTA in HCC tumor tissues were positively correlated with the salvage-to-de novo ratio (Fig. 2e).

To further confirm the metabolic interplay linking cancer methionine metabolism and T-cell exhaustion in HCC, we analyzed single-cell RNA sequencing data to further interrogate the relationship. Among 5112 single-cell transcriptomes data from four HCC patients(Supplementary Table 6)^18^, we identified 534 malignant cells and associated T cells. We divided the cancer cells into salvage-high and de novo-high by their corresponding expression and found that three out of four tumors were composed predominantly from salvage-high cells while one tumor was enriched with de novo-high cells (Fig. 2f and Supplementary Fig. 4). T cells derived from salvage-dominant tumors and the de novo-dominant tumor differ in their transcriptomic profiles, and, specifically, the T cells originated from salvage-dominant tumors showed a significantly higher degree of T-cell exhaustion (*p* = 0.04, Fig. 2f, g). Notably, there is a trend of increasing methionine metabolism and higher DNA methyltransferase activity in T cells derived from salvage-dominant tumors as comparing to those from de novo-dominant tumors (Fig. 2g). As SAM is a well-documented methyl group donor, our data suggest an active interaction between tumor methionine metabolic status and associated T lymphocytes via SAM production. Taken together, the single-cell findings nicely confirmed the findings reported above from analyzing the bulk expression data.

When analyzing somatic copy number alterations (SCNA), we found that, in the TIGER-LC cohort, there are multiple genes within the salvage pathway (*MTAP, SRM, SMS*, and *APIP*) and the de novo pathway (*MTR, BHMT*, and *BHMT2*) that show a very strong association between the mRNA alterations observed and the SCNA and the distribution of significant molecular alterations correlated with the changes of SAM/MTA abundance and T-cell exhaustion level in tumors (Supplementary Fig. 5a, and Supplementary Table 7). Importantly, these SCNA molecular alterations were not detected in matched non-tumor liver tissues, confirming they were tumor-specific changes. Similar results were observed in both LCI and TCGA-LIHC cohorts (Supplementary Table 7), whereas the prevalence of molecular alternations varied across the different cohorts (Supplementary Fig. 5b). In the salvage pathway, alterations involving *SRM, SMS*, and *AMD1* were more prevalent in the Asian population, whereas changes associated *ENOPH1* were the highest in the Caucasian population. For the de novo pathway, gain or upregulation of *GNMT*, *MTR* and *AHCY* were the most prevalent features in all three cohorts. Collectively, these results testify that genes involved in methionine metabolic pathway are preferentially amplified in HCC, are associated with specific methionine metabolic reprogramming alterations, which may drive HCC progression.

### Methionine metabolite levels as potential biomarkers of immune activity and HCC patient survival

**Fig. 3:**
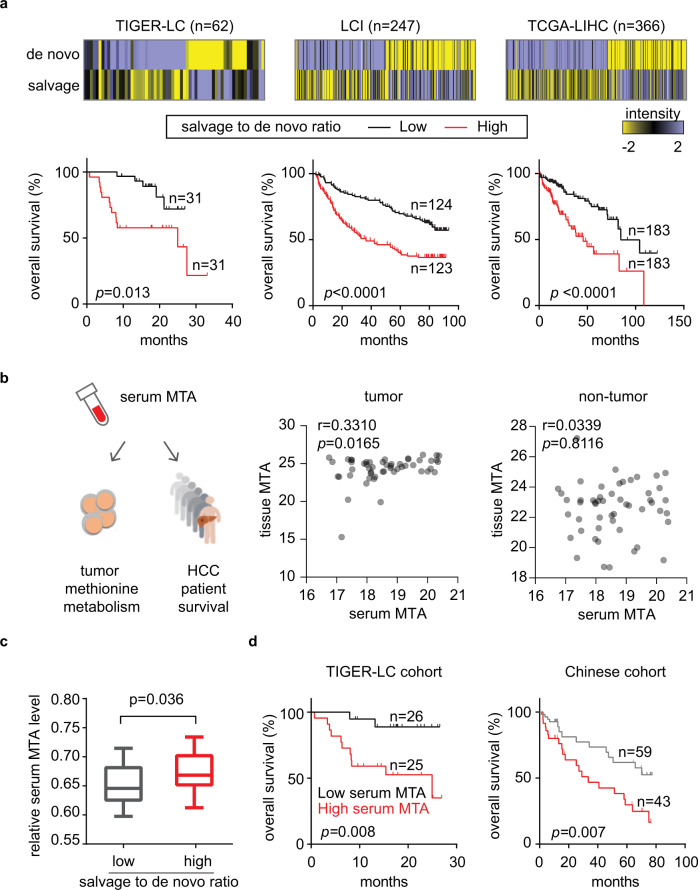
Methionine metabolic reprogramming and serum MTA level predict HCC patient survival. **a** Kaplan–Meier survival curves of patients with high versus low salvage-to-de novo ratio. The median value of the salvage-to-de novo ratio is used to divide patient into two groups and the *P* value is computed using a two-sided log-rank test. Heatmap attached to each cohort summarizes the intensity of salvage pathway and de novo pathway in individual HCC tumor. **b** The relationship of serum MTA level with the MTA level in matched tumor (right) and non-tumor (left) tissues. (*n* = 52) Serum MTA level is adjusted to corresponding serum methionine level for this and subsequent analysis. Correlation coefficient and P values are based on two-sided Spearman’s rank correlation coefficient test. **c** Serum MTA level corresponding to tumor methionine metabolic status. Comparison of serum MTA was performed on patients with available serum metabolome data and methionine metabolic status was defined by the median value of tumor salvage-to-de novo ratio (*n* = 20 in low salvage-to-de novo group, *n* = 26 in high salvage-to-de novo group). The boxplots summarize the distribution of serum MTA level in tumors with high- or low- tumor salvage-to-de novo ratio. The minimum and maximum values are described by the extension of whiskers; the medium value is indicated by the middle line within box, and the 25th and 75th percentiles are indicated by the edges of box. Statistical significance is determined by two-sided independent *t* test. **d** Serum MTA predicts HCC patient survival. HCC patients from TIGER-LC cohorts (*n* = 51) and from LCI cohort (n = 102) are separated by median serum MTA level and Kaplan–Meier survival analysis are shown here with two-sided log-rank *p* value. (Supplementary Data 4). Source data are provided as a Source Data file.

To study the clinical relevance of methionine metabolic changes in HCC, we examined the association between methionine recycling and HCC patient survival. In all three cohorts, we found that higher tumor salvage-to-de novo ratio was strongly associated with a significant worse patient outcome (Fig. 3a). We then examined whether methionine metabolites could be quantified in patients’ serum and thus be used as the biomarkers of tumor methionine metabolism and of patient survival. We performed metabolomic analysis of tumor tissues and corresponding serum samples from 51 HCC cases in the TIGER-LC cohort^11^. Although SAM could not be detected in patients’ serum, MTA levels could be quantified among the 616 metabolites identified. We found a trend of increasing tumor MTA content in patient with high serum MTA (Pearson’s *r* = 0.3310, *p* = 0.0165), but such relationship was not observed between MTA levels of serum and with non-tumor tissues (Fig. 3b). High serum MTA levels were associated with activation of methionine salvage pathway in HCC tumors (Fig. 3c) and worse patient survival (Fig. 3d, left panel), as hypothesized. Importantly, the survival predictions inferred from the serum MTA levels were validated in an independent cohort of 102 Chinese HCC patients^16^ (Fig. 3d, right panel, and Supplementary Data 4). Taken together, these results suggest the promising role of serum MTA levels as an additional biomarker of the overall survival of HCC patients.

### Tumor methionine metabolism determines tumorigenesis and CD8+ T-cell function

**Fig. 4:**
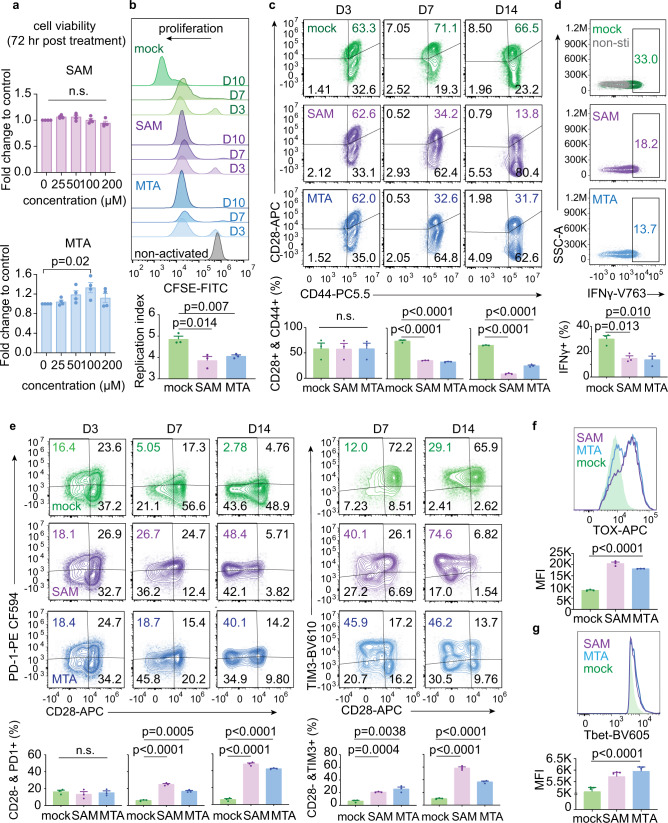
SAM and MTA treatment promotes dysfunction of human CD8+ T cells in vitro. **a** The viabilities of activated CD8+ T cells after exposing to indicated concentrations of SAM or MTA for 72 h. Data are presented as mean ± S.D.; *n* = 4 independent biological experiments. *P* value was calculated using a one-way ANOVA test with Tukey’s multiple comparisons test for subgroup comparison. **b** Representative histogram of CFSE-labeled human peripheral CD8+ T cells underwent indicated treatments and indicated time points. The effects of different treatments on cell proliferation are compared using the proliferation index estimated by FlowJo (lower panel). Data are presented as mean ± S.D. and *p* value between mock and each treatment was calculated using two-sided independent *t* test. (*n* = 3 independent biological experiments). **c** Representative expressions of CD44 and CD28 of human CD8+ T cells underwent SAM, MTA or mock control treatment for 3, 7, and 4 days. Expressions of CD44 and CD28 on CD3+ CD4− CD8+ CD45+ cell were analyzed and the average fraction of cells positive for CD44 and CD28 at various time points are shown at the bottom panel. Bar, mean; error bars, S.D.; *n* = 3 independent biological experiments. Statistical significance was assessed by two-sided independent *t* test. **d** SAM/MTA treatment attenuated interferon-gamma secretion after PMA/ionomycin stimulation. Representative interferon-gamma expression of cells CD3+ CD4− CD8+ CD45+ T underwent indicated treatments were shown. The average fraction of cells with increasing interferon-gamma expression is shown below. Bar, mean; error bars, S.D.; *n* = 3 independent biological experiments. *P* value between mock and each treatment was calculated using two-sided independent *t* test. **e** Representative expressions of PD1, TIM3, and CD28 of human CD8+ T cells underwent SAM, MTA, or mock control treatment for 3, 7, and 14 days. The average fraction of CD3+ CD4− CD8+ CD45+ T cells positive for CD28, PD1, and TIM3 at various time points are shown at the bottom panel. Data are presented as mean ± S.D. and *p* value between mock and each treatment was calculated using two-sided independent *t* test. (*n* = 3 independent biological experiments). **f**, **g** SAM/MTA treatments promote the expressions of TOX and Tbet in CD8+ T cells. Representative histograms and median fluorescence intensity (MFI) of TOX and Tbet in CD3+ CD8+ TOX+ T cells **f** and CD3+ CD8+ Tbet+ T cells **g** were shown. Bar, mean; error bars, S.D.; *n* = 3 independent biological experiments. *P* value was calculated using a one-way ANOVA test with Tukey’s multiple comparisons tests. Source data are provided as a Source Data file.

To further investigate the roles of tumor methionine metabolism in affecting tumor immunity and tumorigenesis, we first examined the biological effects of SAM and MTA on CD8+ T cells. Freshly isolated human CD8+ T cells from healthy donors were stimulated by anti-CD2/CD3/CD28 beads, treated with SAM or MTA, and examined their impacts on cell viability, proliferation, and functional status. By exposing CD8+ T cells with various doses of SAM or MTA for 3 days, we found that these metabolites up to 200 µM did not cause acute cytotoxicity of CD8+ T cells (Fig. 4a). Next, we used SAM 100 µM or MTA 100 µM, ~1/10 of estimated average tumor SAM/MTA abundance^19^, to further clarify the effects of these two methionine metabolites on CD8+ T cells. Using carboxyfluorescein diacetate succinimidyl ester (CFSE), a cell permeable dye used in measuring cell proliferation, we found that the proliferation potential of CD8+ T cells was not altered by SAM or MTA at Day 3 after stimulation, but a significant reduction in cell proliferation induced by SAM or MTA treatment was observed at Day 7 and later time point (Fig. 4b). Importantly, we observed time-dependent changes of T-cell activation and exhaustion markers in CD8+ T cells treated with SAM or MTA (Fig. 4c, e). At day 3, the degree of T-cell activation, characterized by co-expression of CD28 and CD44, and T-cell dysfunction, characterized by positive PD1 or TIM3 but negative CD28, were similar among CD8+ T cells with SAM or MTA compared with mock treatment. However, the fraction of activated T cells was significantly decreased in SAM or MTA-treated lymphocytes at Day 7 and Day 14 compared with mock-treated T cells (Fig. 4c), while the presence of T-cell dysfunction increased significantly with SAM or MTA treatment (Fig. 4e). SAM or MTA also suppressed cytokine production; on average, the intensity of interferon-gamma was ~50% reduced in cells exposed to SAM or MTA compared with an activated control (Fig. 4d). Importantly, we examined the expressions of TOX and Tbet, two important exhaustion-related transcription factors^4,20^, and found that the expressions of TOX and Tbet were significantly higher in CD8+ T cells treated with SAM and MTA compared with control (Fig. 4f, g). Collectively, our data indicate that SAM and MTA attribute to the progression of T-cell dysfunction in vitro.

**Fig. 5:**
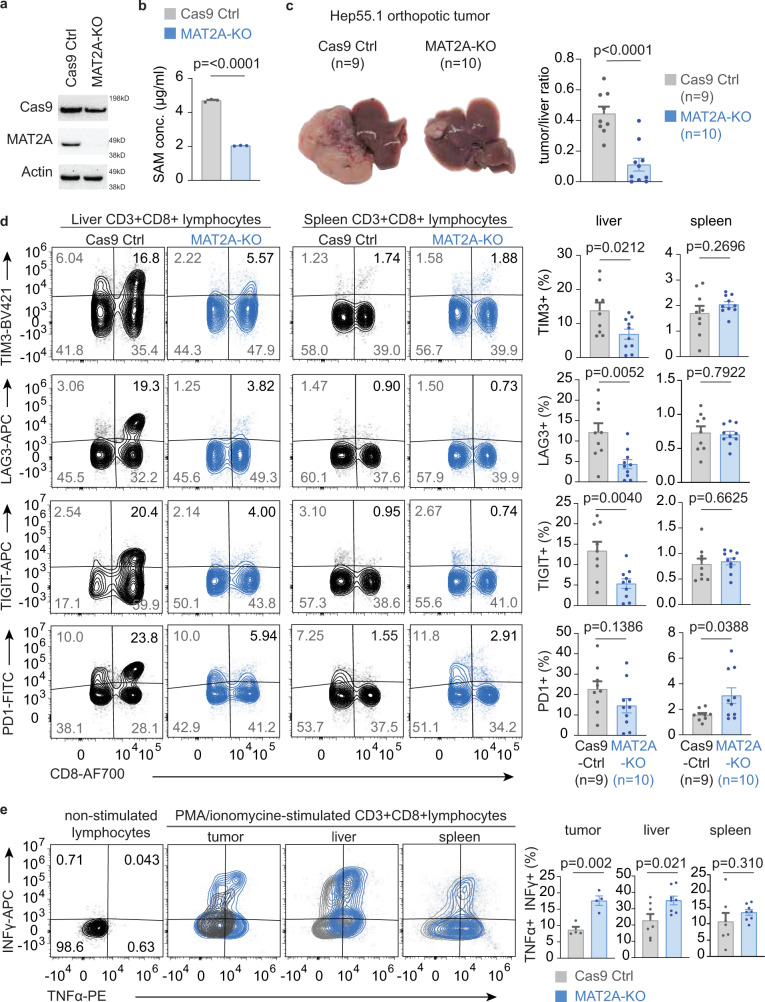
Knockout of MAT2A reduces SAM production and suppressive in vivo tumorigenesis and T-cell dysfunction in HCC. **a** Representative immunoblot of Hep-55.1 cells with stable MAT2A-KO and control of three independent experiments. Corresponding molecular weight markers of each blot were labeled at the right edge of image and the original full scan images could be find in the Source Data. **b** Intracellular concentration of SAM of Hep-55.1 cells with stable MAT2A-KO and control. Bar, mean; error bar, S.D. *n* = 3. Statistical significance is determined by two-sided independent *t* test. **c** Representative livers of mice carrying Hep-55.1 tumors with and without MAT2A-KO (left). The average ratio of tumor-to-liver weight is shown at the left panel (*n* = 9 in Cas9-Ctrl and *n* = 10 in MAT2A-KO). Bar, mean; error bar, S.D. Statistical significance is determined by two-sided independent *t* test. **d** Representative expressions of TIM3, LAG3, TIGIT, and PD1 on CD8+ T cells isolated from tumor-carrying livers and spleens. The average fraction of cells positive for each markers from liver or spleen were shown at the right two panels (*n* = 9 in Cas9-Ctrl, *n* = 10 in MAT2A-KO). Bar, mean; error bar, S.D. Statistical significance is determined by two-sided independent *t* test. **e** Representative expressions of interferon-gamma (INFγ) and tumor necrosis factor- alpha (TNFα) of CD8+ T cells after PMA/ionomycin stimulation. The average fraction of CD8+ T cells positive for INFγ and TNFα is summarized by bar plots. Lymphocytes obtained from tumors (*n* = 4 biological independent samples from Cas9-Ctrl and from MAT2A-KO tumors), liver (*n* = 7 biological independent samples from Cas9-Ctrl mice, *n* = 8 in biological independent samples MAT2A-KO) and spleen (*n* = 7 biological independent samples in Cas9-Ctrl, *n* = 8 biological independent samples in MAT2A-KO) were stimulated with PMA/ionomycin for 4 h and stained for cytokine expressions. Bar, mean; error bar, S.D. Statistical significance is determined by two-sided independent *t* test. Source data are provided as a Source Data file.

We used Hep-55.1 syngeneic HCC model to further investigates the roles of tumor methionine metabolism in tumorigenesis and tumor immunity. MAT2A is a key enzyme of SAM/MTA production, and preferential activation of MAT2A in HCC tumors has been characterized^21^. We used clustered regularly interspaced short palindromic repeats (CRISPR)-Cas9 system to generate Hep-55.1 cells with stable knockout (KO) of MAT2A (Fig. 5a). We used enzyme-linked immunosorbent assay to validate that SAM content was significantly reduced in MAT2A-KO Hep-55.1 cells (Fig. 5b). We also examined the impact of MAT2A-KO on cell viability but did not observe significant differences of the in vitro proliferation rate among Hep-55.1 cells with and without MAT2A-KO (Supplementary Fig. 6b). Orthotopic HCC tumors were established by direct injection of Hep-55.1 cells with MAT2A-KO or Hep-55.1 with stable expression of Cas9-control vector into mouse livers. At the experimental endpoint, intrahepatic tumor, liver, and spleen were harvested for further analysis. Knockout of MAT2A significantly reduced the growth of Hep-55.1 liver tumor (*p* < 0.0001, Fig. 5c and Supplementary Fig. 6c). We isolated CD8+ T lymphocytes from tumor-associated liver tissues and spleens to study how tumor methionine metabolic status affects the function of CD8+ T cells. The fractions of CD8+ T cells positive for exhaustion markers, such as TIM3, LAG3, and TIGIT, were significantly depleted in MAT2A-KO tumors compared to control (Fig. 5d). Notably, we did not observe corresponding changes among the CD8+ T cells isolated from the spleens of mice carrying MAT2A-KO and Cas9-Ctrl liver tumors. Interestingly, we found that the fraction of CD8+ T lymphocytes expressed exhaustion markers increased with an increasing weight of Cas9-Ctrl tumors, but such relationship was not observed in liver carrying MAT2A-KO tumors (Supplementary Fig. 6e). To further confirm the function exhaustion phenotypes, we examined the responses of CD8+ T cells isolated from tumors, tumor-associated livers, and spleens to phorbol myristate acetate (PMA)/ionomycin stimulation. As shown in Fig. 5e, we found that CD8+ T cells isolated from MAT2A-tumors or their surrounding liver tissues showed a significantly better PMA/ionomycin-induced cytokine production compared with corresponding cells isolated form control tumors, but such differences were not observed in CD8+ T cells isolated from spleens. Collectively, our results indicate that tumor methionine metabolism plays key roles in tumorigenesis and T-cell immunity of HCC, and production of methionine metabolites, SAM and MTA, attributes to the progression of T-cell dysfunction in vitro and in vivo.

### SAM/MTA metabolites reprogram chromatin accessibilities of CD8+ T cells linking to T-cell dysfunction

**Fig. 6:**
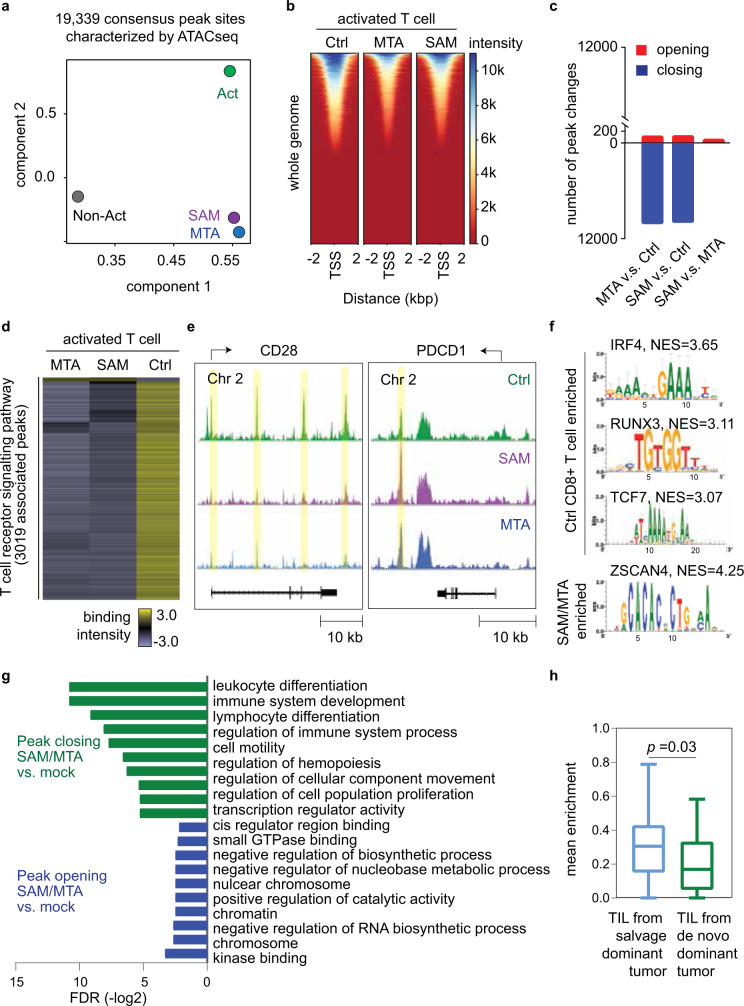
SAM and MTA treatment reduced the global chromatin accessibilities of activated CD8+ T cell. **a** Principal component analysis of peak accessibility in SAM-, MTA-, and mock-treated activated CD8+ T cells, as well as non-activated CD8+ T cells. Dot represented the consensus peaks of CD8+ T cells underwent indicated treatment (*n* = 3 in each treatment condition). **b** Chromatin accessibility heatmap of SAM-, MTA-, and mock-treated activated CD8+ T cells. **c** Number of peak changes by treatments. (FDR < 0.05). **d** Heatmap of accessibility intensity in 3019 loci associated with T-cell signaling pathway. **e** ATAC-seq coverage at *CD28* and *PDCD1*. Peaks were highlighted in yellow. **f** Sequence motifs enriched in the open chromatin regions of mock-treated or SAM/MTA-treated activated CD8 T cells. **g** Pathway analysis of the most variable peaks associated with SAM/MTA treatment (FDR < 0.05). **h** SAM/MTA-induced chromatin changes correlated with T-cell transcriptome changes in clinical HCC tumors. The differentially expressed ATAC-seq peaks (FDR < 0.05, fold change >1.5) and the most differentially expressed genes in HCC tumors of TIGER-LC cohort (FDR < 0.05) were compared. After excluding tumor-specific genes, we identified 12 genes as SAM/MTA-driven T-cell genes, defined as genes showed the most variable chromatin changes in SAM/MTA-treated CD8+ T cells and, coincidentally, were significantly upregulated in salvage-dominant HCC tumors. The expressions of SAM/MTA-driven T-cell genes were examined in HCC-infiltrating T cells (GEO125449). The boxplots summarize the distribution of the mean expression of SAM/MTA-driven T-cell genes obtained from salvage-dominant tumors (*n* = 154 T lymphocytes from three patients) to de novo-dominant tumors (*n* = 21 T lymphocytes from one patient). The minimum and the maximum values are described by the extension of whiskers; the medium value is indicated by the middle line within box, and the 25th and 75th percentiles are indicated by the edges of box. Statistical significance is determined by two-sided independent *t* test. ATAC-sequencing data are available at the GEO repository under Study Accession GSE166213. Source data are provided as a Source Data file.

As SAM is an important cofactor in regulating chromatin process^22^, we used an assay for transposase-accessible chromatin with high-throughput sequencing (ATAC-seq) to assay the genome-wide chromatin accessibility of T cells during activation and SAM/MTA treatment. ATAC-seq libraries were generated from SAM- or MTA-treated cells, as well as from vehicle-treated activated-control and non-activated control. We called peaks on individual replicates, identified consensus peak regions, merged, and normalized these data with DiffBind and DESeq2^23,24^. Among a total of 19,339 consensus peak sites, we found substantial chromatin changes that differentiated SAM- and MTA-treated cells from mock-treated activated T cells and non-activated T cells (Fig. 6a). Notably, in comparison with non-activated T cells, the epigenetic profiles of SAM- or MTA-treated cells showed a relatively closer correlation with activated T cells, in line with our in vitro findings showing that the effects of SAM or MTA did not result in preventing T cells being activated initially, but instead, inhibited effector T-cell function after activation, possibly driving them to an exhausted post-activation state.

Next, we focused on the changes between SAM- or MTA-treated and mock-treated activated T cells. The distributions of transposase-accessible sites across the whole genome were not significantly altered in SAM- or MTA-treated cells, but SAM or MTA treatment led to a general reduction of peak intensity, suggesting a closed chromatin status, comparing with control, with less changes between SAM- and MTA-treated cells (Fig. 6b, c). We also examined the T-cell activity genes and found that a majority of the loci associated with T-cell receptor signaling, such as *CD28*, showed decreased binding intensity (Fig. 6d, e, left panel). Interestingly, we found an increasing binding intensity associated with an intergenic peak near the *PDCD1* locus in SAM- or MTA-treated cells, which is consistent with a similar chromatin change in exhausted T cell^25,26^. Further functional exploration was performed by conducting pathway analysis on the genes that showed most variable chromatin accessibilities following SAM or MTA treatment. Transcription factor enrichment analysis showed that *IRF4, RUNX3*, and *TCF7* were among the top transcription factor with specific motifs being inhibited by SAM or MTA treatment, whereas *ZSCAN4*, a transcription factor associated with induction of cellular senescence^27^, was found to be enriched in SAM or MTA-treated cells (Fig. 6f). Moreover, as shown in Fig. 6g, genes with decreasing chromatin accessibilities following SAM or MTA treatment were mainly enriched in pathways associated with lymphocyte proliferation and differentiation, whereas genes involving chromosome structure were among those with increasing accessibilities. At last, we examined whether the in vitro chromatin changes of CD8+ T cells correlated with the T-cell transcriptome affected by tumor methionine status in clinical HCC samples. In comparison of T-cell ATAC-seq and HCC bulk tumor transcriptome, we identified 12 genes that showed most variable changes in SAM or MTA-induced chromatin accessibilities in CD8+ T cells and most significant upregulation in salvage-dominant HCC tumors (Fig. 6h and Supplementary Table 8). Notably, we found corresponding transcriptomic changes in HCC-infiltrating T cells. The mean expression of SAM/MTA-driven T-cell genes were significantly enriched in T cells obtained from salvage-dominant tumors than de novo-dominant tumors (*p* = 0.03). In summary, our data indicated that SAM and MTA treatments reduce global chromatin accessibilities in CD8+ T cells, leading to T-cell dysfunction.

## Discussion

Emergent evidence suggests that intratumoral mechanisms of metabolite exchange in the TME act cooperatively to support tumor growth while impairing immunity^28^. For example, reprogramming of glucose and lactate metabolism in tumor cells may provide a cross-feeding mechanism between cancer cells and immune community^6,29,30^. Cancer cells in a hypoxic environment preferentially undergo anaerobic glycolysis that metabolizes glucose to lactate, which not only can be uptaken and utilized by well-oxygenated cancer cells to fuel mitochondrial metabolism, but can also inhibit the antitumor function of effector T cells and macrophages^28^. Tryptophan avidity and the release of the tryptophan metabolite kynurenine by cancer cells have also been shown to impair antitumor effector T cells in TME^31,32^. Moreover, SAM has been shown to regulate hepatocyte growth and differentiation, whereas alterations of SAM biosynthesis and metabolism may contribute to the development of HCC^33–35^. It hence has become evident that cancer cells effectively activate unique tumor metabolic process by utilizing specific metabolites to overcome nutrient deprivation and immune surveillance in the TME.

In this study, we discovered that tumor methionine metabolism exerts significant impacts on tumorigenesis and two tumor methionine metabolites, SAM and MTA, may promote immune dysfunction in HCC. We revealed a role of SAM and its downstream metabolite, MTA in regulating CD8+ T-cell function. We showed that SAM and MTA directly reprogram the global chromatin accessibility of T cells, tilting it towards the function exhaustion of T cells in TME. Mechanistically, we found that the reprogramming of methionine recycling machinery may be preferentially selected during tumorigenesis via SCNA of methionine metabolic genes, thereby resulting in an accumulation of SAM/MTA in HCC. The anti-inflammatory effect of SAM and MTA on hepatic macrophages induced by lipopolysaccharide simulation has been reported previously^36^. Interestingly, previous studies showed a reduced hepatic biosynthesis of SAM in all forms of chronic liver injury^37^. In contrast, we found that the levels of SAM and MTA in tumors are significantly higher than that of paired non-tumor tissues. The increasing requirement of methionine metabolism in HCC tumors suggests that SAM and MTA may play a different role in development versus maintenance of HCC.

Immune checkpoint inhibitors (ICIs) have consistently been shown to produce sustain tumor control with improving clinical survival in HCC patients^38,39^. However, most cases do not respond to ICIs and mechanisms underlying therapeutic resistance remain unclear. Here, we showed that the degree of T-cell exhaustion in HCC patients is highly related to the SAM/MTA levels in TME, and tumor methionine metabolic activity was linked to degree of T-cell dysfunction in tumor-associated liver tissues in vivo. From a translational perspective, our results have two main future ramifications: (1) Our data suggest that reprogramming of methionine metabolism may be one of the tumor resistance mechanisms to ICI. Thus, inhibiting tumor methionine metabolism may represent a potential strategy to enhance ICI response in HCC. (2) Bearing in mind that effective biomarker predicting ICI responsiveness is still lacking, here we showed that serum MTA levels are associated with the degree of T-cell exhaustion in HCC tumors and with patient survival. This suggests that serum MTA levels could potentially serve as a biomarker predicting ICI efficacy in patients with HCC, which should be further explored in the future.

## Methods

### HCC cohorts

We identified 675 HCC samples and 347 tumor-free liver samples with available genetic and clinical data from three independent published cohorts, in detail, that included 62 tumor and 58 associated non-tumor tissues from the TIGER-LC cohort (GSE76297)^11^, 247 tumors and 239 associated non-tumor tissues from the LCI cohort (GSE14520)^40^ and 366 tumor and 50 associated non-tumor tissues the TCGA-LIHC cohort (TCGA Research Network: http://cancergenome.nih.gov/)^41^. In addition, we also obtained the transcriptomic profile of liver tissues with chronic hepatitis (GEO84346)^42^ and acute hepatitis (GSE57214, GSE13440, and GSE82771)^43–45^ from the NCBI GEO Repositories for comparison.

### Identification and computing expression of exhaustion signature in bulk samples

From the published single-cell RNA sequencing data set that profiled 5063 HCC-associated T lymphocytes (GSE98638), we used transcriptome changes to define exhausted CD8+ T-cell clusters and identified a list of 82 genes that were specifically upregulated in the exhausted T-cell cluster as exhaustion signature (Supplementary Data 1)^10^. To delineate the expressions of exhaustion signatures in bulk tumor samples, we normalized the expressions of each gene within the exhaustion signatures to mean expressions of *CD3D*, *CD3E*, and *CD3G*.

### Feature selection and construction of ES

For feature selection, we used the CD3-normalized expressions of the 82 exhaustion genes as features and focused on the linear combinations of features that could construct the best survival risk prediction model. We used TIGER-LC cohort as the training cohort and used the “Survival Risk Prediction” function of BRB-Array Tool to build the model. In brief, in the TIGER-LC cohort, we developed a supervised principal component model by first identifying a subset of features that were most significantly correlated with patient survival (alpha = 0.001) and validated the prediction of the established model using leave-one-out-cross-validation strategy. Based on above-mentioned procedure, four features, namely *YARS, PKM2, TPI1*, and *MTHFD2*, were selected to build ES, and each feature were further given a weight derived from the correlation of feature and survival (Supplementary Table 2). ES was computed based on the value (*x*_i_) and associated weight (*w*_i_) of the ith feature using the following function:

$${\mathrm{Exhaustion}}\,{\mathrm{score}} = \mathop {\sum}\limits_i {w_ix_i - 12.77237} .$$

### Modeling of T-cell function and tumor methionine metabolism

To model the change of CD8+ T-cell function from bulk transcriptome, we used ES to infer the degree of T-cell exhaustion/dysfunction, and used the geometric mean of *GZMA* and *PRF1*, a metric published by Rooney et al.^12^, to estimate the cytolytic CD8+ T-cell function (referring as CYT).

For methionine metabolism, we focused 11 methionine metabolic genes with known function involving methionine recycling pathways in liver^46–48^. The cumulative expressions of genes involving de novo pathway, namely *GNMT, AHCY, BHMT, BHMT2*, and *MTR*, and salvage pathway, including *AMD1, SRM, SMS, ENOPH1, MTAP*, and *APIP*, were used to represent the activity of each pathway. For bulk samples, the ratio of the expression of salvage pathway to that of the de novo pathway was calculated to model the tumor methionine metabolic status. For single-cell RAN-sequencing data, we used the difference of the salvage pathway expression to the de novo pathway expression to model methionine metabolic reprogramming in HCC cells.

### Tumor and serum metabolome analysis in HCC patients

We identified HCC patients with metabolomic profiling performed in their tumor and paired non-tumor tissues, which included 63 patients in TIGER-LC cohort^11^ and 30 patients in LCI cohort^16^. Because these two studies were carried out at different time points by different platform^49^, there were information of 718 metabolites shown in the TIGER-LC cohort, and it was 469 metabolites reported in the LCI cohort. For both cohorts, the missing value were imputed using the minimum value of each metabolite. To examine whether tumor metabolic alterations affect T-cell function, we first identified 57 patients from TIGER-LC cohort with available information on transcriptome and metabolome. We then evaluated the Spearman rank correlation coefficients (*ρ*) and corresponding *P* value between the ES and the relative tumor/non-tumor ratio of all the metabolite and identified metabolite that showed the highest correlation as our hit. Further investigations on examining how the identified hits, SAM and MTA, present in HCC tumors and how they relate to ETC and tumor methionine metabolism were carried out in TIGER-LC cohort and LCI cohort.

For global serum metabolome analysis, we also used the platform from Metabolon^49^ Inc. for sample preparation and data analysis. In brief, there were 51 patients from TIGER-LC cohort, and 102 patients from Chinese cohort had their serum samples been analyzed (Supplementary Data 4). Serum MTA level was first normalized to serum methionine level and then utilized for subsequent analysis. For serum metabolome studies, informed consent was obtained from patients and the use of serum samples were approved by the Institutional Review Boards of the participating institutes^11,40^.

### Copy number variation of methionine metabolic genes

Among all the HCC cases enrolled in this study, 61 cases from TIGER-LC cohort (GSE76213) and 370 cases from TCGA-LIHC cohort have SNP-array based copy number data, and 76 patients (GSE14322) from LCI cohort have CGH-array based copy number data. The raw copy number data for each probe of above-mentioned patients were obtained and analyzed by Nexus Copy Number 9.0 software (Biodiscovery, Inc.). To examine whether methionine metabolite genes are regulated by copy number changes, we calculated the Pearson correlation value between the medium probe value called by Nexus and the transcriptome data of each gene. For significant copy number alterations, the threshold of medium probe value was set to +0.8 for amplification and −0.8 for deep deletion.

### Analysis of HCC single-cell data sets

We used the single-cell RNA sequencing data set recently reported by our laboratory (GEO125449) to interrogate the interaction of tumor methionine metabolism, tumor biodiversity, and T-cell function. From this data set, we first identified cancer cells and associated T cells from patients with confirmed HCC. The expressions of genes involving methionine recycling pathways (detailed above) were obtained and the intensity differences of these two pathways were calculated. Then, we classified cancer cells by their relative methionine metabolic status; salvage-high type referred to HCC cells with salvage-to-de novo intensity differences above the mean intensity difference of all cancer population, whereas the opposite subgroup was referred as de novo-high type. Above information was then used to build up the composition of each HCC tumor regarding to methionine metabolic status. For T cells, we first applied t-SNE analysis to access the diversity across T-cell transcriptome. Then, we obtained the expressions of exhaustion genes (Supplementary Data 1), methionine metabolic genes, and DNA methyltransferase, namely *DNMT1, DNMT3a*, and *DNMT3b*, in T cells. Each T cells were then grouped according to the methionine metabolic status of their originated tumor and compared by student *t* test.

### Isolation of human CD8+ T cells

Peripheral blood mononuclear cells (PBMC) from health donor were first isolated from buffy coat by density gradient centrifugation using Ficoll-Pagque (Miltenyi Biotec). CD8+ T cells were then isolated from PBMC using CD8 microbeads (Miltenyi Biotec.), and purity was assessed by flow cytometry. Subsequent experiments were carried out after confirming CD8+ T-cell population was >95% pure. For all the experiments, CD8+ cells were cultivated at TexMACS medium (Miltenyi Biotec) at the density of 1 × 10^6^ cells per ml per cm^2^ with additional interleukin-2 supplement (50 IU/mL, Miltenyi Biotec.).

Blood products used in this experiment were obtained from the blood bank of National Health Institute (Bethesda, MD, USA). The experiments were reviewed and approved by the Institutional Review Board of the National Cancer Institute (Bethesda, MD, USA), and informed consent was obtained from donor in accordance with the Declaration of Helsinki.

### In vitro testing of SAM and MTA on human CD8+ T cells

Based on the relationships between the concentration of all amino acids shown in literature^19^ with the corresponding level characterized in our metabolomic study (Supplementary Table 9), we estimated the average concentrations of SAM and MTA in HCC tissues were 1.023 mmol/kg and 1.223 mmol/kg. Therefore, the dose of 100 µM SAM and MTA, ~10% of the level estimated in HCC tissues, were used for most of the in vitro testing. For acute cytotoxicity, we incubated activated CD8+ T cells with various doses of SAM and MTA for 72 h and examined for viability using CellTiter-Glo (Promega). For assessing relative time-dependent effects of SAM and MTA on CD8+ T cells, we used CFSE dilutional stain to tract cell division and flow cytometry to examine the expressions of T-cell markers (detailed in Supplementary Table 10) at indicated time points. Two T-cell exhaustion-associated transcriptional factors, TOX and Tbet, were also examined at Day 8 after activation and SAM/MTA treatment. In brief, CD8+ T cells were stained with CFSE at the concentration of 5 µM, followed by stimulation with anti-CD2/CD3/CD28 beads (Miltenyi Biotec.) at the cell-to-bead ratio of 2:1. We then incubated 1 × 10^6^ stimulated CD8+ T cells with SAM 100 µM or MTA 100 µM or vehicle control. Subfraction of cells in each condition was harvested for detection of CFSE signal at Day 3, 7, and 10. These cells were also examined for the expressions of T-cell surface markers in parallel on Day 3, Day 7, and Day 14. For CFSE test, we also include unstimulated CFSE-labeled cells as a non-dividing control. For detection of INF-γ, cells were exposed to SAM or MTA first for 48 h and stimulated with PMA 50 ng/ml (Sigma) and Ionomycin 1 µg/ml (Sigma) in the presence of monensin-containing protein transport inhibitor (BD) for 5 hours. Then, these cells were collected, fixed by a fixation/permeabilization kit (BD), and proceeded to intracellular staining. In all the above-mentioned experiments, we did not remove the CD2/3/28 beads from CD8+ T cells after initial activation.

All flow cytometry experiments included live/dead staining to discriminate live or dead cells and we applied Fluorescence Minus One control to define positivity of every marker of interest. Flow cytometry data were acquired on an CytoFLEX LX Flow Cytometer with CytExpert v2.3 software and we used flowJo software V10 to analyze the signal captured by flow cytometry. The effects of SAM and MTA were compared specifically within CD3+ CD4− CD8+ CD45+ T cells, and the strategy we used to identify CD3+ CD4− CD8+ CD45+ T cells were demonstrated in Supplementary Figure 6a.

### Sample preparation for ATAC-seq

We conducted ATAC-seq on SAM-treated, MTA-treated activated CD8+ T cells, as well as mock-treated activated CD8+ T cells and non-activated CD8+ T cells. Each condition contains three replicates. For SAM, MTA, and activated-control replicates, CD8+ T cells were activated by anti-CD2/CD3/CD28 beads and incubated with SAM 100 µM or MTA 100 µM or vehicle control right after stimulation for 7 days prior to cryopreservation. For non-activated control, CD8+ T cells were planted on plate overnight and harvested for cryopreservation. All the samples were frozen in Recovery Cell Culture Freezing Medium (Thermo Fisher) and stored at −80 °C before library preparation.

We used the OMNI ATAC-seq protocol introduced by Corces et al.^50^ for library preparation. In brief, 50,000 viable cells were pelleted (500 relative centrifugal force (RCF), 4 °C, 5 min), and lysed by resuspending in 50 µl cold ATAC-resuspension buffer with detergents (10 mM Tris-HCl; 10 mM NaCl; 3 mM MgCl_2_; 0.1% NP40; 0.1% Tween-20, and 0.01% Digitonin) and incubating for 3 min, followed by washing with the same buffer, but without NP40 and Digitonin. The nuclei were pelleted (500 RCF, 4 °C, 10 min) and resuspended in 50 µl transposition mix (1× TD buffer Illumina cat# 15027866), 100 nM transposase (Illumina cat# 15027865), 1× PBS, 0.01% digitonin, 0.1% Tween-20). Transposition was performed at 37 °C for 30 min in a thermomixer with shaking at 1000 r.p.m. After purification of DNA by Zymo DNA Clean and Concentrator-5 kit (cat#D4014), amplification was performed for seven cycles using NEBNext 2× MasterMix (New England Biolabs cat# M0541S). The PCR reaction was purified by double size selection with SPRI beads, to remove any primer dimers (1.3× ratio) and large fragments over 1000 bp (0.5× ratio). Additional cleanups were performed to remove primer dimers, as needed.

### ATAC sequencing, data processing, and differential peak accessibility analysis

After generating ATAC-seq libraries, 12 samples were pooled and sequenced on HiSeq4000 using in a 150 bp/150 bp paired end run. An average of 120 × 10^6^ paired reads was generated per sample. Raw ATAC-seq reads were trimmed and filtered with Cutadapt v1.18 and aligned to hg38 using Bowtie2 v2.2.6. Uniquely aligned reads were identified using picard v2.18.26 MarkDuplicates utility with 72–79% non-duplicates mapped reads among all the samples. Peak from individual samples was called using Genrich (https://github.com/jsh58/Genrich) with the argument -j -y -r -v -d 150 -m 5 -e chrM, chrY -E blacklist_regions.bed to exclude reads from mitochondria and reads from regions on chrY or those that intersected ENCODE blacklisted regions. A total of 135,583 peaks were identified from 12 samples and we used DiffBind^23^ v3.9 (https://bioconductor.org/packages/release/bioc/html/DiffBind.html) to combine and identify consensus peak loci, and to perform differential peak accessibility analysis. Peak annotation was performed using ChIPseeker v3.9^51^, and pathway analysis and motif enrichment analysis were performed on the most variable peak loci among different conditions (Fold change > 1.5, FDR < 0.001) using the Compute Overlaps function in MSigDB (https://www.gsea-msigdb.org/gsea/msigdb/annotate.jsp) and i-*cis*Target^52,53^, respectively.

For visualization, consensus peaks from biological replicates were merged, and peak heat maps were created by deepTools2^54^, and genome coverage plot was generated by IGV v2.7.x^55^.

### Cell culture

Murine HCC cell line, Hep-55.1 (CLS number: 400201), was purchased from Cell Line Service (Eppelheim, Germany) and maintained in Dulbecco’s Modified Eagle Medium (DMEM) supplement with 10% fetal calf serum and 5% l-glutamine.

### Generation of MAT2A-KO cell using CRSPR/Cas9 genome editing

sgRNA targeting ACGAGGCGTTCATTGAGGAGGGG of MAT2A were designed^56^, cloned into lenti-SpCas9 puro (Addgene Plasmid 104994) and packaged using HEK293T ells using Lenti-Pac HIV Expression Packaging Kit (GeneCopia). Hep-55.1 cells were transduced with lenti-SpCas9-2A-Puro (empty vector) or lenti-SpCas9-2A-Puro-MAT2A viral supplemented with 8 µg/mL polybrene for 48 h and selected using 2.5 µg/mL puromycin. Validation was performed by immunoblotting for anti-MAT2A and anti-Cas9, whereas anti-β-actin was included as control (Supplementary Table 10).

### Detection of SAM

SAM level was measured using SAM ELISA kit (Cell Biolabs) according to the instructions provided by the manufacturer’s manual. In brief, 1 × 10^7^ HCC cells were harvested in 1 ml of ice-cold PBS and underwent sonication to harvest intracellular metabolites. Intracellular SAM levels of Hep-55.1 cells with MAT2A-KO and control were then quantified by comparing the ELISA signal of SAM standard.

### In vitro cell proliferation

The xCELLigence RTCA biosensor system (ACEA Biosciences) was utilized to compare the in vitro proliferation rates of Hep-55.1 cells with and without MAT2A-KO. In brief, 3500 cells were seeded to each well of 16-well E-Plate (ACEA Biosciences) and the electrical impedance created in each well were monitored for 96 h. The changes of impedance were displayed as Cell Index.

### Mice experiment

Murine HCC cell line, Hep-55.1, was purchased from Cell Line Service (Eppelheim, Germany) and maintained in DMEM supplement with 10% fetal calf serum and 5% l-glutamine.

Female C57BL/6 mice were obtained from NCI/Frederick (Frederick, MD, USA). All mice were group-housed (five mice per cage) and maintained under a regular light–dark cycle altered every 12 h with free access to water and standard mouse chow. Hep-55.1 cell was examined for Molecular Testing of Biological Materials-Mouse/Rat (LASP Animal Health Diagnostic Laboratory, NCI-Frederick), which evaluated mycoplasma and 15 other pathogens. The animal experiments were reviewed and approved by the NCI-Bethesda Institutional Animal Care and Use Committee (Bethesda, MD, USA).

Liver tumor induction were performed to 8–10-week-old mice. In brief, 3 × 10^5^ Hep-55.1 cells mixed in 20 µL of a 1:1 solution of Matrigel (Corning) and PBS (Thermo Fisher) were injected into the left lobe of liver of anesthetized mice after subcostal laparotomy as described before^57^. In total, 20 mice received tumor implantation and 19 of them (9 in control group and 10 in MAT2A-KO group) survived after this procedure. Tumors were allowed to grow for 24 days and mice were then killed to harvest liver and spleen. Body weight of mice were measured before obtaining the organs. Liver and tumor were weighted, and tumor-to-liver weight ratio was calculated and compared between groups. Lymphocytes for tumor, liver lymphocytes and splenocytes were isolated for flow cytometric analysis. For surface markers and immune checkpoint proteins, cells were labeled with the indicated antibodies for 30 min at 4 °C. For TNFα and INFγ, we first seeded 5 × 10^5^ cells in 100 µL RMPI medium on a 96-well plate. PMA/ionomycin stimulation and intracellular staining for cytokines were proceeded as described in the human T-cell experiments. FlowJo v.10 was used for analyzing the results obtained using flow cytometry. T-cell markers and cytokines were compared within CD3+ CD8+ T cells, and the gaiting strategy were demonstrated in Supplementary Figure 6d.

### Statistical analysis

Unsupervised hierarchical clustering analysis was performed by GENESIS V1.8.1 (IBMT-TUG, Graz, Austria). Cox proportional hazards regression was performed using SPSS statistics Subscription version 1.0.0.1089 (IBM) to compare the prognosis prediction of different features. Pathway analysis was conducted using ingenuity pathway analysis (QIAGEN Bioinformatics). To examine the significance of mean differences between groups, we used two-tailed unpaired or paired Student’s *t* tests for normally distributed data, and non-parametric Mann–Whitney *U* test for non-normally distrusted data. The relationship between two features of interest were determined by Spearman rank correlation test. Kaplan–Meier survival analysis was performed to compare patient survival and the statistical *p* was generated by the Cox-Mantel log-rank test. All the statistics tests were performed using GraphPad Prism V7.0. unless otherwise specified. All *p* values presented were two-sided and a *p* < 0.05 was considered as statistically significant.

### Reporting summary

Further information on research design is available in the Nature Research Reporting Summary linked to this article.

## Supplementary information

Supplementary Information

Descriptions of Additional Supplementary Files

Supplementary Data 1

Supplementary Data 2

Supplementary Data 3

Supplementary Data 4

Reporting Summary

## Data Availability

Public data sets used in this study could be downloaded from NCBI Gene Expression Omnibus (GEO) under the accession number GSE76297, GSE76213, GSE14520, GSE14322, GSE63898, GSE15765, GSE98638, GSE125449, GSE84346, GSE57214, GSE13440, GSE82771, GSE76297, GSE14520, and from TCGA portal (https://portal.gdc.cancer.gov). ATAC-sequencing data are available at the GSE repository under study accession GSE166213. The remaining data supporting the findings of this paper are provided in the article, supplementary information, or are available from the corresponding author upon request. Source data are provided with this paper.

## Source data

Source Data
