# Supplementary material for: Tumor methionine metabolism drives T-cell exhaustion in hepatocellular carcinoma
Source: Nat Commun. 2021 Mar 5;12:1455. doi: 10.1038/s41467-021-21804-1 (PMC7935900; doi:10.1038/s41467-021-21804-1)
Supplement: Supplementary file 1 — Supplementary Information [file 41467_2021_21804_MOESM1_ESM.pdf]

Supplementary Information

Tumor methionine metabolism drives T-cell exhaustion in hepatocellular carcinoma

Man Hsin Hung[1], Joo Sang Lee[2], Chi Ma[3], Laurence P. Diggs[3], Sophia Heinrich[1], Ching Wen Chang[1], Lichun Ma[1], Marshonna Forgues[1], Anuradha Budhu[4], Jittiporn Chaisaingmongkol[5], Mathuros Ruchirawat[5,6], Eytan Ruppin[2], Tim F. Greten[3,4], Xin Wei Wang[1,4*]

1. Laboratory of Human Carcinogenesis, Center for Cancer Research, National Cancer Institute, Bethesda, Maryland, USA
2. Cancer Data Science Lab, National Cancer Institute, National Institutes of Health, Bethesda, Maryland, USA
3. Gastrointestinal Malignancy Section, Thoracic and Gastrointestinal Oncology Branch, Center for Cancer Research, National Cancer Institute, National Institutes of Health, Bethesda, MD 20892, USA.
4. Liver Cancer Program, Center for Cancer Research, National Cancer Institute, Bethesda, Maryland, USA
5. Laboratory of Chemical Carcinogenesis, Chulabhorn Research Institute, Bangkok, Thailand
6. Center of Excellence on Environmental Health and Toxicology, Office of Higher Education Commission, Ministry of Education, Bangkok, Thailand

*Send correspondence to: xw3u@nih.gov

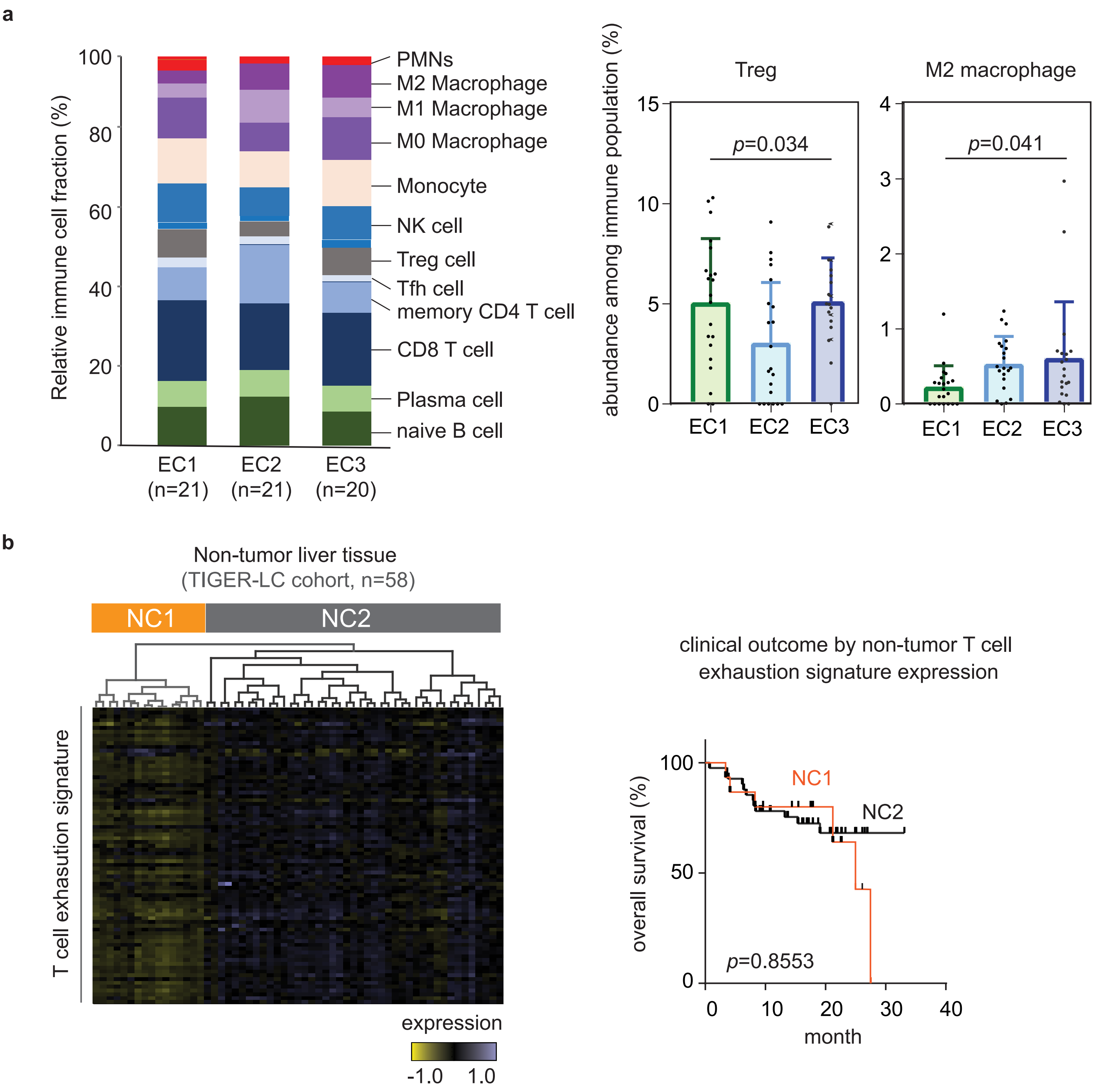

Supplementary Figure 1. T cell exhaustion signature of tumor issues is unique in characterizing immuno-suppressive TME.
(A) The composition of immune community associated with exhaustion clusters. CIBERSORT is applied to infer the content of immune cells, and the average composition of immune cells in different exhaustion clusters are shown on the left panel. Right two panels detail the distribution and the average percentages of T regulatory cell and M2 macrophage among all immune cell population in the three exhaustion clusters (n=21 in EC1, n=21 in EC2, n=20 in EC3). Bar, mean; error bar, S.D. Statistical significance is determined by on one-way ANOVA test.
(B) The expressions of T cell exhaustion signatures in the non-tumor tissues of TIGER-LC cohort (n=58). Kaplan-Meier curve represents the survival outcomes of patients divided according to the non-tumor expressions of T cell exhaustion signature. Statistic significance was determined using a two-sided log rank test.
Source data are provided as a Source Data file.

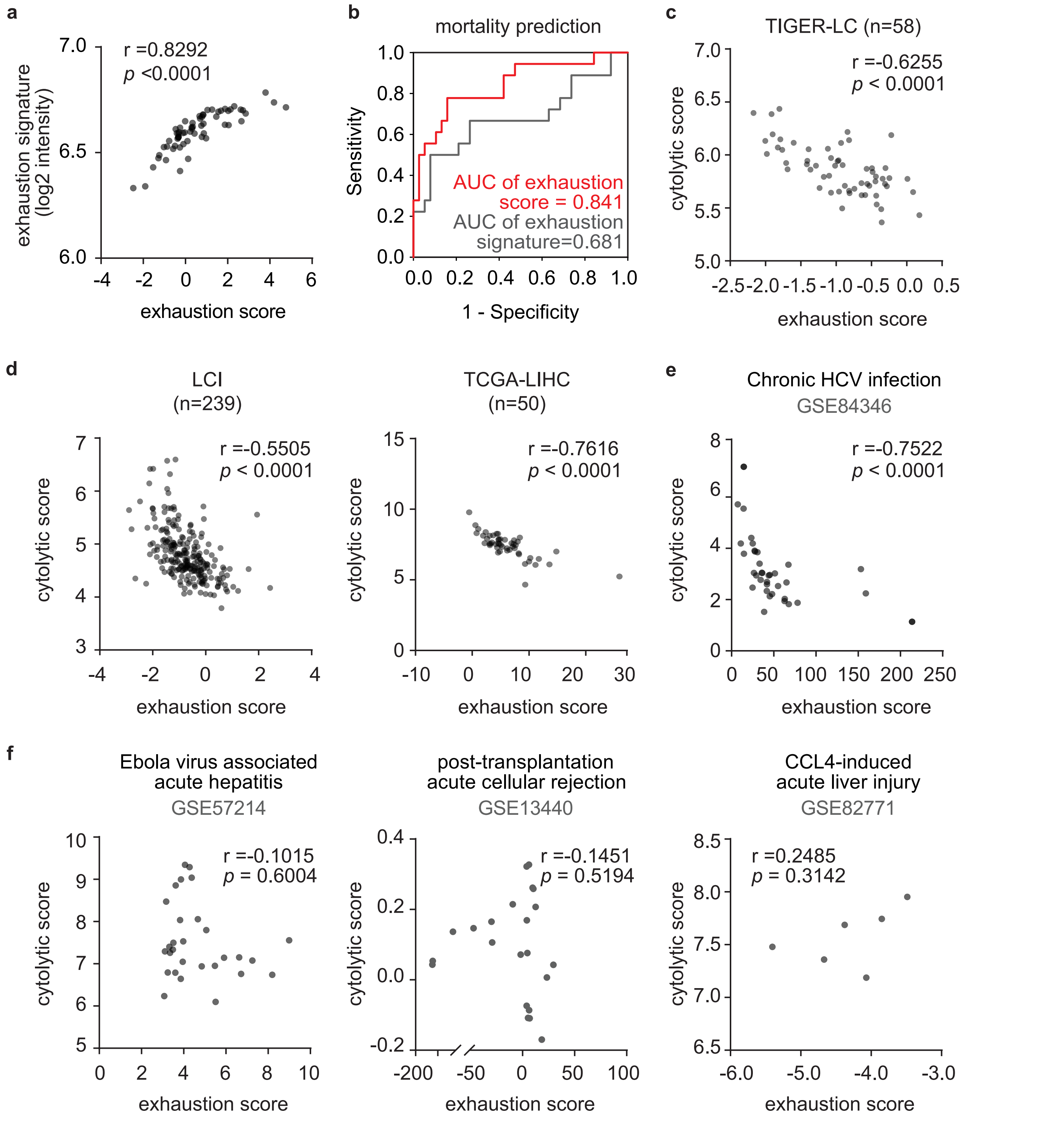

Supplementary Figure 2. Exhaustion score specifically capture the progression of T cell dysfunction in chronic inflammatory conditions with improving survival prediction than the originated T cell exhaustion signature.

(A) The relationship of exhaustion score and the intensity of the original 82 T-cell exhaustion-specific genes. Correlation coefficient and p value are calculated using two-sided Pearson Correlation Analysis.

(B) The performances of exhaustion score and the T-cell exhaustion-specific genes in predicting patient survival in TIGER-LC cohort.

(C-F) The relationship of exhaustion score and cytolytic score in non-tumor cirrhosis tissues obtained from HCC patients (C, D), in liver tissues with chronic hepatitis C infection (E), and in liver tissues with indicated acute infection/inflammation conditions (F). Correlation coefficient and p value are calculated using two-sided Spearman’s rank correlation coefficient test. Source data are provided as a Source Data file.

Supplementary Figure 3

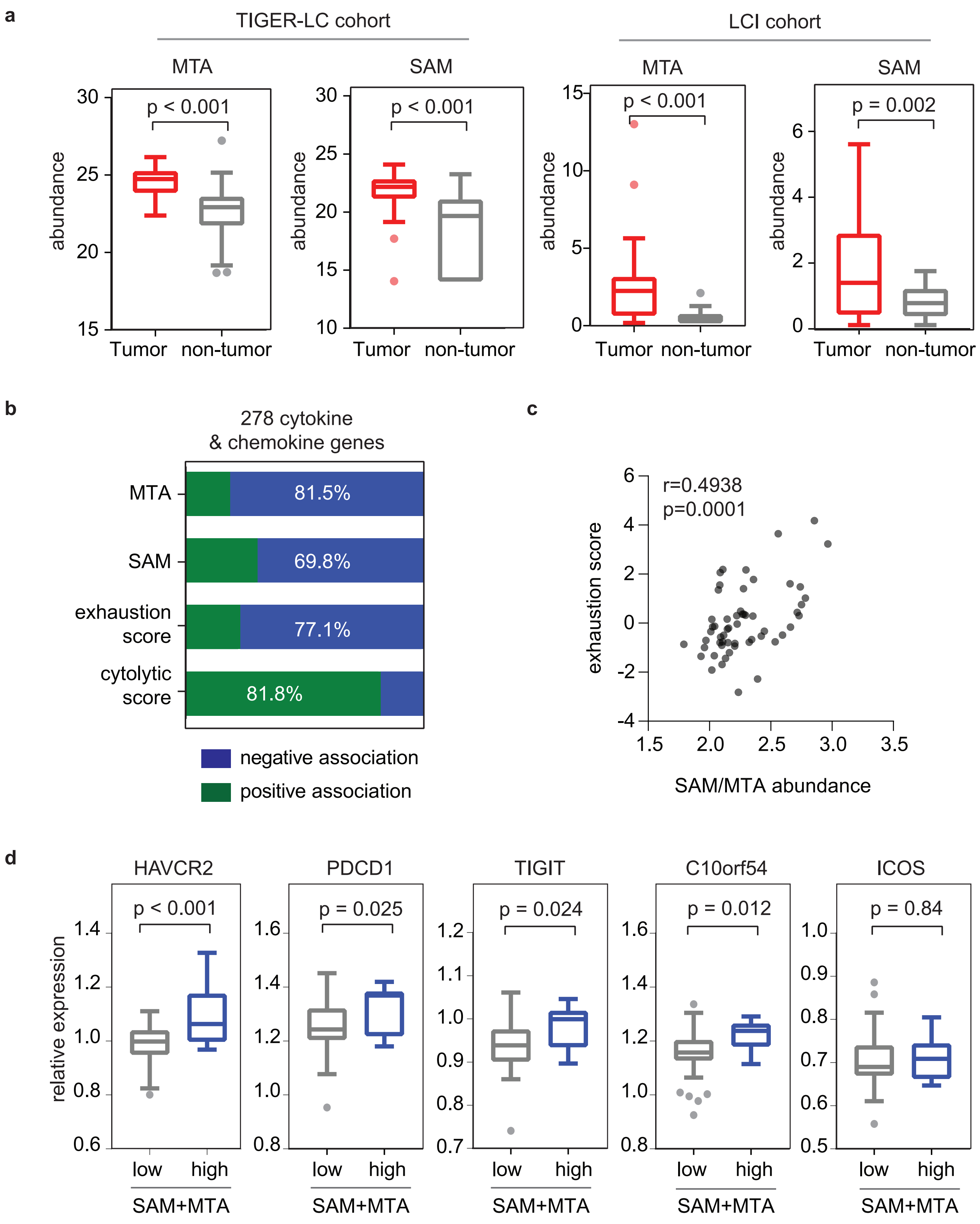

Supplementary Figure 3. Tumor SAM and MTA content are associated with T cell exhaustion status.

(A) Boxplot of the abundance of MTA and SAM in tumor (n=56 in Tiger-LC cohort, n=30 in LCI cohort) and non-tumor tissues (n=63 in Tiger-LC cohort, n=30 in LCI cohort) of are shown. The medium values are indicated by the horizontal line within box and the edges of box depict the interquartile range (IQR). Whiskers extend to data within 1.5x the IQR and the outliers are plotted individually if presented. Statistical significance was determined using a two-tailed independent t test between tumor and non-tumor.

(B) Relationship of global cytokine/chemokine signaling and the levels of MTA, SAM, ES and CS in HCC tumors.

(C) The relationship of tumor ES and SAM/MTA content in TIGER-LC cohort. Correlation coefficient and p value were calculated using two-sided Pearson Correlation Analysis.

(D) Boxplots showing the expressions of immune checkpoint of tumors with high SAM and MTA content (top 20%, n=12) versus the rest of tumor (n=44) in TIGER-LC cohort. The expressions of four negative immune-checkpoints related to ETC and one positive immune-checkpoint, ICOS, related to effector T cell are identified from bulk transcriptomes and adjusted to mean CD3 for analysis. The medium expressions of checkpoints of each subgroup is indicated by the horizontal line within box and the edges of box depict the interquartile range (IQR). Whiskers extend to data within 1.5x the IQR and the outliers are plotted individually. P values are based on two-sided independent t test between groups.

Source data are provided as a Source Data file.

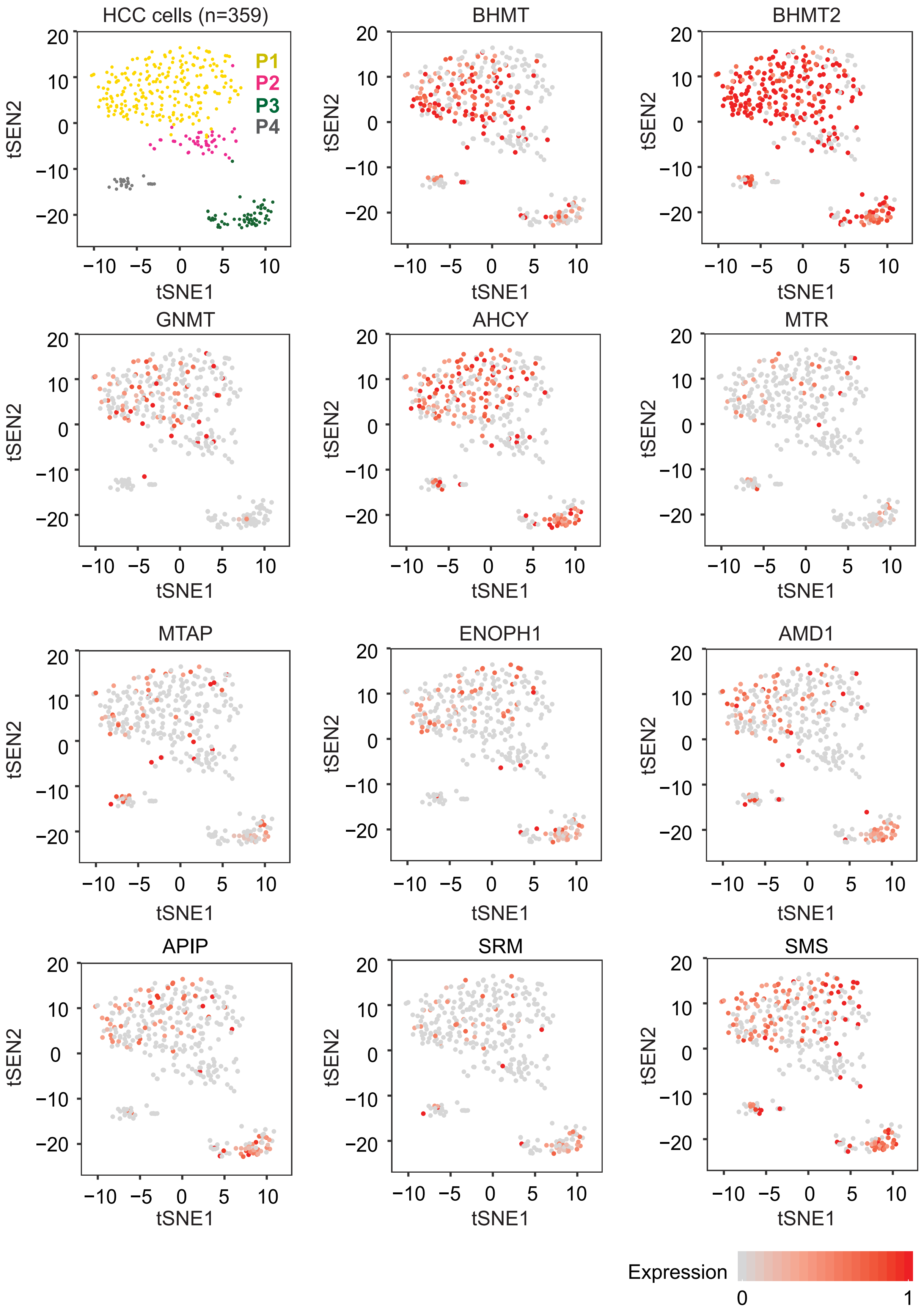

Supplementary Figure 4. Variation of methionine metabolic gene expression in HCC cancer cells
t-SNE plots of 359 malignant cells from four HCC tumors (indicated by colors) and the expressions of genes involving methionine recycling pathways.

Supplementary Figure 5

a

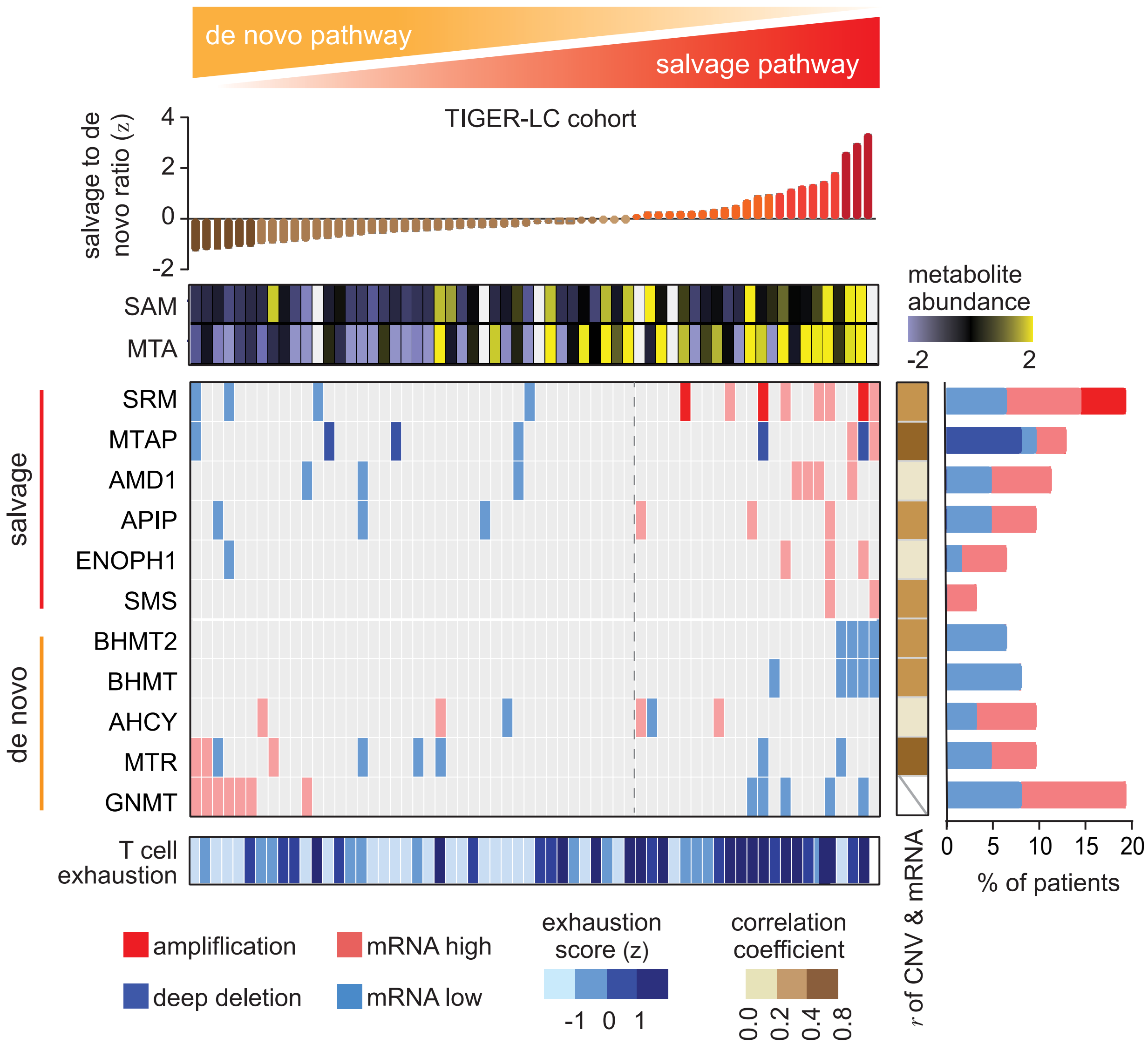

b

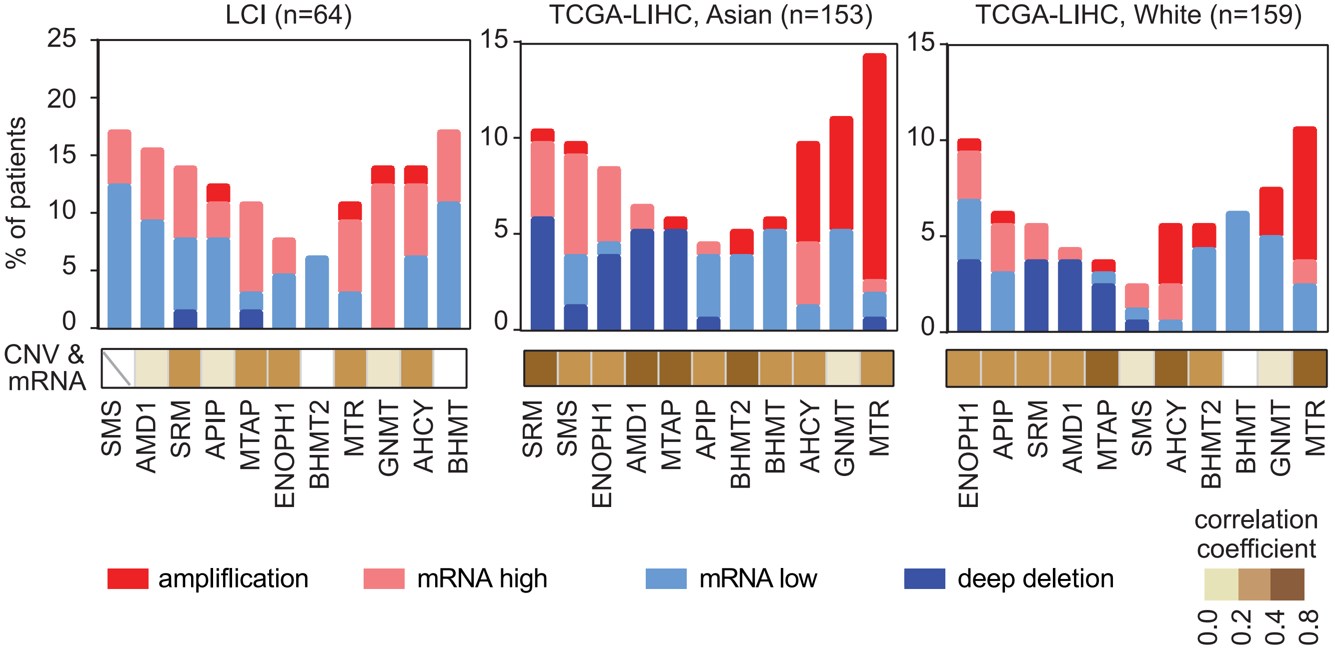

Supplementary Figure 5. Somatic copy-number alterations of methionine metabolic genes drive the dysregulation of methionine recycling machinery in HCC tumors.

(A) The relationship between the dysregulation of methionine recycling pathways and the significant genomic changes are shown alone with the abundance of SAM/MTA and the intensity of T cell exhaustion. Top panel shows the salvage-to-de novo ratio of individual tumor, followed by the tumor SAM and MTA content. The middle panel details the presence of significant copy-number (CN) variations and transcriptomic changes of genes involving methionine recycling machinery in each patient. Dark red bar represents significant CN gain (median probe value > 0.8) with increased gene expression (amplification) and dark blue bar represents significant CN loss (median probe value < 0.8) with suppressed gene expression (deep deletion). Changes of gene expression is represented by the pink bar and light blue bars that indicates increased (z-transformed intensity > 1.8, mRNA high) or decreased (z-transformed intensity < -1.8, mRNA low) in the tumor. For each gene, the correlation of CN and expression and the frequency of significant genetic alterations were summarized over the right panel (see also Supplementary Table 7).

(B) The genomic alterations of methionine recycling pathways are observed in ethnicity-independent cohort. The frequency of significant genetic changes (upper panel) and the correlation of CN and mRAN expression (lower panel) of LCI cohort, TCGA-LICH Asian cohort, and TCGA-LICH Caucasian were shown.

Supplementary Figure 6

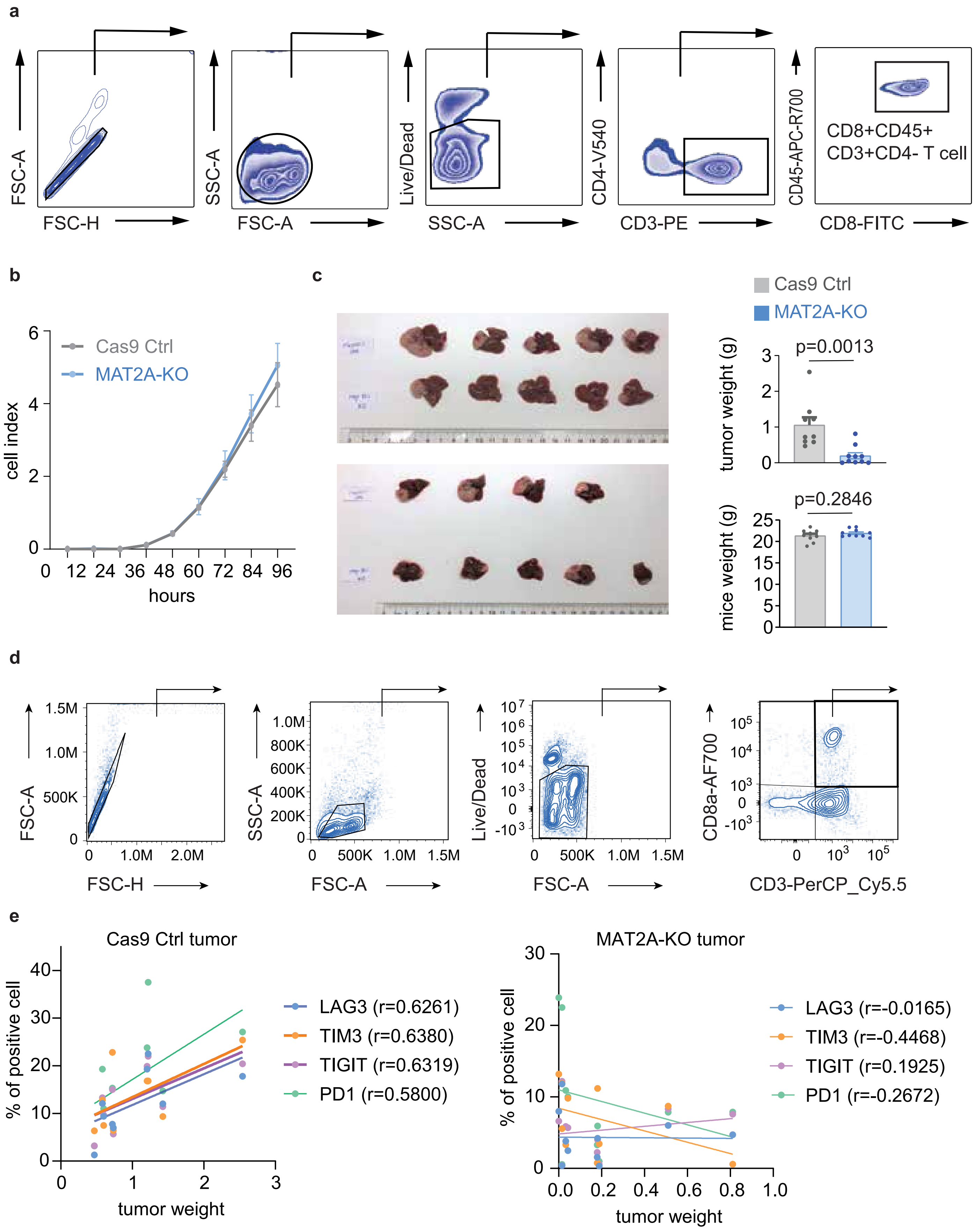

Supplementary Figure 6. MAT2A impacts tumorigenesis and T cell function in HCC.

(A) The gating strategy used in human T cell experiments (associated with Figure 4b-e).

(B) The *in vitro* growth curves of MAT2A-KO Hep-55.1 cells and Cas9-Ctrl Hep-55.1 cells. Dot, mean; error bar, S.D. (n=3)

(C) The pictures of livers from mice carrying MAT2A-KO and Cas9-Ctrl tumors.
The average weight of mice and tumor were summarized at the right panels. Bar, mean; error bar, S.D. Statistical significance is determined by two-sided independent t test.

(D) The gating strategy for analyzing lymphocytes isolated from tumor, liver and spleen (associated with Figure 5d-e).

(E) Relationship of tumor weight and the fraction of CD8+ T cells with positive immune checkpoint expression of associated tumor. Correlation coefficient was calculated using Peason Correlation analysis.
Source data are provided as a Source Data file.

Supplementary Table 1. Clinical characterities of HCC patients belongs to different exhausion classes

Characteristics	EC1 (n=21)	EC2 (n=21)	EC3 (n=20)	*p* value*
**Mediam age (range)**	57 (21-67)	54 (40-72)	55 (40-76)	0.899
**Male gender**	19 (90.5)	14 (73.7)	10 (58.8)	0.077
**Clinical TNM stage, N(%)**				0.6
I	7 (35.0)	5 (26.3)	2 (11.8)	
II	7 (35.0)	11 (57.9)	9 (52.9)	
III	4 (20.0)	2 (10.5)	4 (23.5)	
IV	2 (10.0)	1 (5.3)	2 (11.8)	
missing data	1	2	3	
**Child_Pugh score**				0.345
<7	12 (92.3)	14 (73.7)	10 (71.4)	
≥7	1 (7.7)	5 (26.3)	4 (28.6)	
missing data	8	2	6	
**Elevated AFP, N(%)**	9 (45.0)	12 (57.1)	9 (52.9)	0.734
**HBV status, N(%)**				0.959
AVR_CC	5 (29.4)	4 (25.0)	4 (30.8)	
CC	7 (41.2)	7(43.8)	4 (30.8)	
Not-infected	5 (29.4)	5 (31.3)	5 (38.5)	
missing data	4	5	7	
**HCV status, N(%)**				0.548
Positive	3 (15.8)	5 (31.3)	3 (21.4)	
Negtive	16 (84.2)	11 (68.8)	11 (78.6)	
missing data	2	5	6	

Abbreviation: AFP, α-fetoprotein; HBV, hepatitis B virus; AVR_CC, active viral replication and chronic carrier; CC, chronic carrier; HCV, hepatitis C virus.

* Chi-squared test

Supplementary Table 2. Hazard ratio and feature weight of exhaustion signature genes

Feature	Hazard Ratio	Parametric p-value*	Weight
PKM	882.748	0.0000038	3.253772
YARS	804.974	0.0002512	2.298095
TPI1	1393.355	0.0006863	2.259203
MTHFD2	332.284	0.000054	2.110235
TOX	285.814	0.0003209	NA
CSF1	275.242	0.0024622	NA
TNFRSF9	4020.815	0.0036193	NA
DUSP4	277.464	0.0104375	NA
HLA-DMA	331.59	0.0112247	NA
HAVCR2	1225.948	0.0115781	NA
RGS1	12.898	0.0163673	NA
WARS	878.917	0.0173483	NA
PRDX5	74.304	0.0226153	NA
IFI35	66.798	0.0244391	NA
SYNGR2	108.037	0.0278526	NA
TNFRSF1B	161.362	0.0392245	NA
GPR56	145.656	0.0499883	NA
PRDM1	448.948	0.055	NA
RGS2	8.386	0.081	NA
FKBP1A	33.716	0.086	NA
PARK7	31.625	0.092	NA
RALGDS	62.224	0.096	NA
FUT8	36.752	0.101	NA
TRAFD1	35.875	0.102	NA
CD63	18.753	0.109	NA
CD2BP2	48.758	0.11	NA
UBE2F	67.878	0.123	NA
HLA-DRA	22.51	0.127	NA
LAYN	707.855	0.129	NA
CD27-AS1	64.482	0.144	NA
ITM2A	10.594	0.149	NA
CXCR6	0.001	0.151	NA
IGFLR1	72.384	0.171	NA
STAT3	8.991	0.173	NA
NAB1	20.86	0.181	NA
ID3	60.181	0.189	NA
CTSD	7.06	0.194	NA
PHLDA1	0.119	0.196	NA
RAB27A	12.562	0.211	NA
CHST12	25.128	0.221	NA
IL2RB	57.758	0.25	NA
TIGIT	119.819	0.264	NA
VAPA	6.625	0.277	NA
IFNG	433.625	0.286	NA

DFNB31	13.397	0.289	NA
GZMB	42.671	0.29	NA
PRKAR1A	4.37	0.312	NA
NDFIP2	8.674	0.324	NA
CD27	185.112	0.337	NA
BST2	3.348	0.358	NA
CCND2	28.893	0.369	NA
AFAP1L2	20.112	0.369	NA
ACP5	6.787	0.391	NA
SNAP47	5.424	0.399	NA
PDCD1	8.657	0.401	NA
MS4A6A	7.317	0.425	NA
MYO1E	3.278	0.435	NA
ENTPD1	8.25	0.446	NA
CCL3	8.236	0.479	NA
HMGN3	3.33	0.486	NA
CXCL13	1.874	0.509	NA
MYO7A	4.434	0.527	NA
SARDH	0.394	0.542	NA
SNX9	2.873	0.566	NA
LAG3	5.618	0.587	NA
CTIF	3.217	0.616	NA
LINC00299	0.315	0.626	NA
PRF1	6.467	0.627	NA
ICOS	0.136	0.657	NA
LYST	0.464	0.661	NA
ITGAE	2.051	0.713	NA
MTHFD1	0.74	0.767	NA
SIRPG	4.578	0.801	NA
CD38	1.726	0.822	NA
AKAP5	0.528	0.829	NA
VCAM1	0.848	0.843	NA
GALM	0.771	0.866	NA
CREM	1.486	0.883	NA

*p value was estimated using univariant Cox proportional hazard regression for survival.

Supplementary Table 3. Mutivariate Cox proportional hazards regression analysis of overall survival in TIGER-LC cohort

	univariant		multivariant	
Factor	HR (95% CI)	*p**	HR (95% CI)	*p**
Child Class B vs. A	3.65 (1.15- 11.57)	0.028	2.71 (0.71- 10.30)	0.144
Chaisaingmongkol's molecular class		0.014		0.242
C1 vs. C3	3.41 (1.26-9.25)		2.54 (0.53- 12.09)	
C2 vs. C3	0.51 (0.11-2.41)		0.89 (0.07- 12.35)	
TNM stage III-IV vs. I-II	3.25 (1.09- 9.66)	0.034	3.27 (0.78- 13.75)	0.105
Exhauscion score	2.718 (1.80- 4.10)	<0.001	3.27 (1.85- 5.79)	<0.001

Abbrevviation: HR, hazard ratio; CI, confidence interval.

*p value was estimated using univariant or multivariant Cox proportional hazard regression for survival.

Supplementary Table 4. Pathway analysis of the variable genes associated with exhaustion score

	Ingenuity Canonical Pathways	-log(p-value)*
immune related	Primary Immunodeficiency Signaling	7.75
	Th1 and Th2 Activation Pathway	7.03
	T Cell Receptor Signaling	6.99
	Th1 Pathway	6.78
	Natural Killer Cell Signaling	6.67
	Th2 Pathway	6.39
	iCOS-iCOSL Signaling in T Helper Cells	4.75
	Calcium-induced T Lymphocyte Apoptosis	4.42
	Role of NFAT in Regulation of the Immune Response	3.92
	B Cell Development	3.61
	CTLA4 Signaling in Cytotoxic T Lymphocytes	3.48
	CD28 Signaling in T Helper Cells	3.36
	Cytotoxic T Lymphocyte-mediated Apoptosis of Target Cells	2.56
	Crosstalk between Dendritic Cells and Natural Killer Cells	2.53
	CCR3 Signaling in Eosinophils	2.4
	T Helper Cell Differentiation	2.33
	CCR5 Signaling in Macrophages	2.28
	Regulation of IL-2 Expression in Activated and Anergic T Lymphocytes	2.22
	IL-8 Signaling	2.17
	B Cell Receptor Signaling	2.09
	Hematopoiesis from Pluripotent Stem Cells	2.09
metabolism	Pyrimidine Deoxyribonucleotides De Novo Biosynthesis I	3.48
	Glycolysis I	3.13
	Methionine Degradation	2.78
	Salvage Pathways of Pyrimidine Ribonucleotides	2.63
	Cysteine Biosynthesis III (mammalia)	2.57
cell cycle and DNA repair	Cell Cycle Control of Chromosomal Replication	11.3
	Role of BRCA1 in DNA Damage Response	8.04
	Mitotic Roles of Polo-Like Kinase	7.27
	Estrogen-mediated S-phase Entry	6.84
	Role of CHK Proteins in Cell Cycle Checkpoint Control	5.94
	DNA damage-induced 14-3-3σ Signaling	5.1
	Mismatch Repair in Eukaryotes	4.65
	Cell Cycle: G2/M DNA Damage Checkpoint Regulation	4.43
	Cell Cycle: G1/S Checkpoint Regulation	4.35
	ATM Signaling	4.05
	Cyclins and Cell Cycle Regulation	3.97
	Cell Cycle Regulation by BTG Family Proteins	2.19
others	GADD45 Signaling	6.2
	tRNA Charging	4.05
	Phospholipase C Signaling	4.02
	VEGF Family Ligand-Receptor Interactions	2.77
	Tec Kinase Signaling	2.76
	RAN Signaling	2.55
	G-Protein Coupled Receptor Signaling	2.08
	Gαi Signaling	2.08
	Nitric Oxide Signaling in the Cardiovascular System	2.04
	Oncostatin M Signaling	2

* p value was calculated using a Right-Tailed Fisher's Exact Test

Supplementary Table 5. List of metabolites that were significantly correlated with exhausion score

Metabolite	Correlation coefficient	P value*	FDR
5-methylthioadenosine (MTA)	0.444	0.0006852	0.04
S-adenosylmethionine (SAM)	0.437	0.0008407	0.04
erythronate*	0.433	0.0009633	0.04
lactate	0.428	0.0010931	0.04
alpha-hydroxyisocaproate	0.42	0.0013957	0.0416
beta-hydroxyisovalerate	0.419	0.0014482	0.0416
N(1)-acetylspermine	0.412	0.001765	0.0463
arabitol	0.41	0.0018647	0.0463
docosadienoate (22:2n6)	0.389	0.0032465	0.0629
erucate (22:1n9)	0.384	0.0037333	0.0646
tryptophan	0.384	0.0036813	0.0646
eicosenoate (20:1n9 or 11)	0.357	0.0072254	0.085
leucylglutamine*	0.35	0.0085945	0.0907
leucylglycine	0.35	0.0085129	0.0907
phenylalanylalanine	0.346	0.0093603	0.0953
ophthalmate	-0.345	0.0095984	0.0957
mannose	-0.346	0.0094194	0.0953
guanidinosuccinate	-0.348	0.0088295	0.0919
uridine	-0.35	0.0084454	0.0907
N-methylpipecolate	-0.351	0.0083251	0.0907
palmitoyl-linoleoyl-glycerophosphocholine (2)*	-0.351	0.0082589	0.0907
succinylcarnitine	-0.356	0.0073794	0.0855
taurocholenate sulfate	-0.364	0.0061425	0.076
1-arachidonoylglycerophosphoethanolamine*	-0.365	0.0059816	0.0756
glycochenodeoxycholate	-0.365	0.0060015	0.0756
6-phosphogluconate	-0.366	0.005728	0.0748
propionylcarnitine	-0.366	0.005728	0.0748
1-methylhistamine	-0.369	0.0054473	0.0738
fumarate	-0.37	0.0053115	0.0737
glucuronate	-0.371	0.0051787	0.0737
N6-methyladenosine	-0.378	0.004336	0.0649
adenosine 2'-monophosphate (2'-AMP)	-0.379	0.0042324	0.0647
glycerol 3-phosphate (G3P)	-0.379	0.0042324	0.0647
ribulose/xylulose 5-phosphate	-0.38	0.0040881	0.0647
taurolithocholate 3-sulfate	-0.38	0.0040598	0.0647
methylsuccinate	-0.381	0.0039759	0.0647
thiamin (Vitamin B1)	-0.383	0.0037795	0.0646
3-(N-acetyl-L-cystein-S-yl) acetaminophen	-0.388	0.0033285	0.0629
hexenedioylcarnitine*	-0.392	0.0030169	0.0602
pyridoxal	-0.401	0.0023421	0.0526
3-phosphoglycerate	-0.407	0.002022	0.0468
betaine aldehyde	-0.408	0.0019365	0.0463
malate	-0.41	0.0018718	0.0463
gamma-glutamylglutamine	-0.423	0.0012806	0.04

glucose	-0.423	0.0012656	0.04
N-acetyl-1-methylhistidine*	-0.424	0.0012607	0.04
stearoyl sphingomyelin	-0.43	0.0010566	0.04
N6-succinyladenosine	-0.431	0.001011	0.04
3-methylglutarylcarnitine (2)	-0.436	0.0008793	0.04
N1-Methyl-2-pyridone-5-carboxamide	-0.46	0.0004115	0.0351
glycocholenate sulfate*	-0.474	0.0002624	0.0335
glutamine	-0.479	0.0002236	0.0335
3-methylglutarylcarnitine (1)	-0.559	1.08E-05	0.00775
unidentified metabolites			
X - 15678	0.482	0.0002015	0.0335
X - 15472	0.392	0.0029685	0.0602
X - 14314	0.377	0.0044495	0.0652
X - 15497	0.361	0.006594	0.0802
X - 22102	0.358	0.0070858	0.0848
X - 13529	0.355	0.0076096	0.0867
X - 12339	-0.342	0.0102167	0.1
X - 10457	-0.369	0.0053384	0.0737
X - 23379	-0.386	0.0035539	0.0646
X - 12681	-0.395	0.0028018	0.0592
X - 12026	-0.398	0.0025581	0.0557
X - 11261	-0.409	0.0019039	0.0463
X - 12953	-0.426	0.0011789	0.04
X - 16397	-0.431	0.0010171	0.04
X - 12014	-0.444	0.0006852	0.04
X - 16581	-0.446	0.0006382	0.04
X - 12093	-0.458	0.0004402	0.0351
X - 12095	-0.463	0.0003769	0.0351
X - 16580	-0.472	0.0002802	0.0335
X - 12125	-0.496	0.0001252	0.0335

* P value was calculated using two-tailed Spearman Correlation Test

Supplementary Table 6. Clinical information of HCC patients involving single cell study (n=4)

Patient characteristics	number (%)
median age (range)	64 (41-74)
male gendar	3 (75%)
Stage III-IV disease	3 (75%)
Etiology	
fatty liver	1 (25%)
chronic Hepatitis C	2 (50%)
unspecified	1 (25%)

Supplementary Table 7. Correlation of mRNA level and copy number variations of methionine metabolic genes

Cohort	TIGER-LC (n=61)		LCI (N=64)		TCGA-LIHC, Asian (n=153)		TCGA-LIHC, White (n=159)	
gene	Spearman r (95% CI)	*p* value*	Spearman r (95% CI)	*p* value*	Spearman r (95% CI)	*p* value*	Spearman r (95% CI)	*p* value*
MTAP	0.749 (0.6095- 0.8436)	< 0.0001	0.3293 (0.0835- 0.5373)	0.0079	0.5529 (0.4283- 0.6569)	<0.0001	0.5176 (0.3897- 0.6258)	<0.0001
SRM	0.3514 (0.1039- 0.5579)	0.0051	0.2753 (0.02417- 0.4937)	0.0277	0.4756 (0.3387- 0.5929)	<0.0001	0.2728 (0.1178- 0.4149)	0.0005
SMS	0.2456 (-0.01202- 0.4726)	0.0544	NA	NA	0.2068 (0.0450- 0.3580)	0.0103	-0.0476 (-0.2062- 0.1135)	0.5516
APIP	0.3423 (0.08445- 0.5573)	0.0085	0.0631 (-0.1928 - 0.3109)	0.6206	0.3261 (0.1720- 0.4647)	<0.0001	0.3491 (0.2001- 0.4823)	< 0.0001
ENOPH1	0.1482 (-0.1152- 0.3921)	0.2544	0.2991 (0.0479- 0.5147)	0.0172	0.2679 (0.1094- 0.4131)	0.0008	0.2986 (0.1453- 0.4378)	0.0001
AMD1	0.1534 (-0.1099- 0.3965)	0.238	0.0552 (-0.2003- 0.3037)	0.6647	0.4562 (0.3165- 0.5765)	<0.0001	0.3854 (0.2400- 0.5138)	<0.0001
BHMT	0.4585 (0.2286- 0.6400)	0.0002	-0.1073 (-0.3505- 0.1495)	0.3988	0.3222 (0.1677- 0.4612)	<0.0001	0.1426 (-0.0180-0.296)	0.073
BHMT2	0.4514 (0.2201- 0.6346)	0.0002	-0.1148 (-0.3572- 0.1421)	0.3663	0.4332 (0.2905- 0.5571)	<0.0001	0.2703 (0.1151- 0.4126)	0.0006
MTR	0.7001 (0.5404- 0.8111)	<0.0001	0.3478 (0.1041- 0.5520)	0.0049	0.3731 (0.2234- 0.5056)	<0.0001	0.4718 (0.3371- 0.5875)	<0.0001
AHCY	0.0204 (-0.2398- 0.2779)	0.8757	0.347 (0.1033-0.5514)	0.005	0.3216 (0.1671- 0.4607)	<0.0001	0.5873 (0.4715- 0.6832)	<0.0001
GNMT	NA	NA	0.1548 (-0.1019- 0.3922)	0.2219	0.0344 (-0.1297- 0.1965)	0.6734	0.0916 (-0.0695- 0.2482)	0.2507

NA, copy number data not avalible

* p value was calculated based on two-tailed Spearman correlation analysis

Supplementary Table 8. SAM/MTA-affected genes

Gene ID	Fold change*
ANXA2	1.886792453
SLC39A10	1.666666667
TPM4	1.587301587
TES	1.5625
ARL4C	1.515151515
NCAPG2	1.492537313
TNFRSF11A	1.470588235
PIP4K2A	1.369863014
MFGE8	1.315789474
MTF2	1.282051282
SSH2	1.265822785
KCMF1	1.162790698

* bewteen salvage dominant to de novo dominant tumor

Supplementary Table 9. Abundance of amino acids in non-tumor tissues (TIGER-LC cohort)

amino acid	mean abundance in non-tumor liver tissues
asparate	1.4713
threonine	1.6317
serine	1.9324
aspragine	1.917
glutamate	1.0779
glutamine	1.2027
glycin	1.4254
alanine	1.4569
valine	1.8666
methionine	2.8884
isoleucine	2.1256
leucine	2.0417
tyrosine	1.8385
phenylalanine	2.1506
ornithine	3.349
lysine	2.3949
histidine	1.4895
tryptophane	1.8797
arginine	0.8614

Supplementary Table 10. Details of antibodies ultilized in flow cytometry and western blotting study

Antibody	identifier	source	dilution
PE Mouse Anti-Human CD3	Clone HIT3A; Cat # 555340	BD Biosciences	1:20
V450 Mouse Anti-Human CD4	Clone RPA-T4; Cat# 560346	BD Biosciences	1:100
FITC Mouse Anti-Human CD8	Clone RPA-T8; Cat#555366	BD Biosciences	1:20
APC-R700 Mouse Anti-Human CD45	Clone HI30; Cat#566041	BD Biosciences	1:100
PerCP-Cy^{TM}5.5 Anti-Human CD44	Clone G44-26; Cat#560531	BD Biosciences	1:100
APC Mouse Anti-HumanCD28	Clone CD28.2; Cat#559770	BD Biosciences	1:20
PE-CF594 Mouse Anti-Human CD279 (PD-1)	Clone EH12.1, Cat# 565024	BD Biosciences	1:50
PE-CF594 Mouse Anti-Human TIM-3 (CD366)	Clone 7D3, Cat#565560	BD Biosciences	1:50
BV786 Mouse Anti-Human IFN-Y	Clone 4S.B3; Cat#563731	BD Biosciences	1:50
TOX Antibody, anti-human/mouse, APC, REAfinity™	Clone REA473; Cat# 130-118-474	Miltenyi Biotec	1:50
Brilliant Violet 605™ anti-T-bet Antibody	Clone 4B10; Cat# 644817	BioLegend	1:50
PerCP/Cyanine5.5 anti-mouse CD3 Antibody	Clone 17A2; Cat#100218	BioLegend	1:200
Alexa Fluor® 700 anti-mouse CD8a Antibody	Clone 53-6.1; Cat#100730	BioLegend	1:1000
FITC anti-mouse CD279 (PD-1) Antibody	Clone 29F1A12; Cat#135214	BioLegend	1:200
APC anti-mouse CD223 (LAG-3) Antibody	Clone C9B7W; Cat# 125210	BioLegend	1:200
BV421 Mouse Anti-Mouse CD366 (TIM-3)	Clone 5D12; Cat#747626	BD Biosciences	1:200
BV650 Mouse Anti-Mouse TIGIT	Clone 1G9 ; Cat# 744213	BD Biosciences	1:200
PE Rat Anti-Mouse TNF	Clone MP6-XT22;	BD Biosciences	1:100
APC Rat Anti-Mouse IFN-gamma	Clone XMG1.2; Cat#554413	BD Biosciences	1:100
MAT2A Antibody	Cat# NB110-94158	Novus Biologicals, LLC	1:1000
Mouse monocloncal anti-beta-actin	Cat#A5316	Sigma-Aldrich	1:1000
Cas9 (7A9-3A3) Mouse mAb	Cat #14697S	CELL SIGNALING TECHNO	1:10000
