# Supplementary material for: Tumor methionine metabolism drives T-cell exhaustion in hepatocellular carcinoma
Source: Nat Commun. 2021 Mar 5;12:1455. doi: 10.1038/s41467-021-21804-1 (PMC7935900; doi:10.1038/s41467-021-21804-1)
Supplement: Supplementary file 2 — Descriptions of Additional Supplementary Files [file 41467_2021_21804_MOESM2_ESM.pdf]

Descriptions of Additional Supplementary Files

Supplementary Data 1

Description: CD8+ T cell exhaustion signature.

Supplementary Data 2

Description: List of the differentially expressed genes among exhaustion classes

Supplementary Data 3

Description: Correlation of cytokine/chemokine genes with the abundance of SAM/MTA and T cell function

Supplementary Data 4

Description: Serum metabolome analysis. 5-methylthioadenosine (MTA) and methionine levels.
